# Gene expression modules in primary breast cancers as risk factors for organotropic patterns of first metastatic spread: a case control study

**DOI:** 10.1186/s13058-017-0881-y

**Published:** 2017-10-13

**Authors:** Katherine Lawler, Efterpi Papouli, Cristina Naceur-Lombardelli, Anca Mera, Kayleigh Ougham, Andrew Tutt, Siker Kimbung, Ingrid Hedenfalk, Jun Zhan, Hongquan Zhang, Richard Buus, Mitch Dowsett, Tony Ng, Sarah E. Pinder, Peter Parker, Lars Holmberg, Cheryl E. Gillett, Anita Grigoriadis, Arnie Purushotham

**Affiliations:** 1 School of Cancer Studies, CRUK King’s Health Partners Centre, King’s College London, Guy’s Campus, London, SE1 1UL UK; 2 Institute for Mathematical and Molecular Biomedicine, King’s College London, Hodgkin Building, Guy’s Campus, London, SE1 1UL UK; 3 NIHR Comprehensive Biomedical Research Centre at Guy’s and St Thomas’ NHS Foundation Trust and King’s College London, London, WC2R 2LS UK; 4 Research Oncology, King’s College London, Faculty of Life Sciences and Medicine, Guy’s Hospital, London, SE1 9RT UK; 5 Cancer Epidemiology Unit, King’s College London, Guy’s Hospital, Great Maze Pond, London, SE1 9RT UK; 6 Cancer Bioinformatics, King’s College London, Innovation Centre, Cancer Centre at Guy’s Hospital, London, SE1 9RT UK; 7 Breast Cancer Now Research Unit, Innovation Centre, Cancer Centre at Guy’s Hospital, King’s Health Partners AHSC, King’s College London, Faculty of Life Sciences and Medicine, London, SE1 9RT UK; 8 Division of Oncology and Pathology, Department of Clinical Sciences, Lund University, Lund, Sweden; 9 CREATE Health Strategic Center for Translational Cancer Research, Lund University, Lund, Sweden; 10 Key Laboratory of Carcinogenesis and Translational Research, Ministry of Education of Beijing, Beijing, People’s Republic of China, Laboratory of Molecular Cell Biology and Tumor Biology, Department of Anatomy, Histology and Embryology, Peking University Health Science Center, Beijing, People’s Republic of China; 11 The Breast Cancer Now Toby Robins Research Centre, The Institute of Cancer Research, London, UK; 12 Richard Dimbleby Department of Cancer Research, Randall Division of Cell and Molecular Biophysics, King’s College London, Guy’s Campus, London, SE1 1UL UK; 13 UCL Cancer Institute, Paul O’Gorman Building, University College London, London, WC1E 6DD UK; 14 London Research Institute, Lincoln’s Inn Fields, London, WC2A 3LY UK; 15 Uppsala University, Department of Surgical Sciences, Uppsala University Hospital, 751 85 Uppsala, Sweden

**Keywords:** Breast cancer, Metasynchronous metastases, Gene expression pattern

## Abstract

**Background:**

Metastases from primary breast cancers can involve single or multiple organs at metastatic disease diagnosis. Molecular risk factors for particular patterns of metastastic spread in a clinical population are limited.

**Methods:**

A case-control design including 1357 primary breast cancers was used to study three distinct clinical patterns of metastasis, which occur within the first six months of metastatic disease: bone and visceral metasynchronous spread, bone-only, and visceral-only metastasis. Whole-genome expression profiles were obtained using whole genome (WG)-DASL assays from formalin-fixed paraffin-embedded (FFPE) samples. A systematic protocol was developed for handling FFPE samples together with stringent data quality controls to identify robust expression profiling data. A panel of published and novel gene sets were tested for association with these specific patterns of metastatic spread and odds ratios (ORs) were calculated.

**Results:**

Metasynchronous metastasis to bone and viscera was found in all intrinsic breast cancer subtypes, while immunohistochemically (IHC)-defined receptor status and specific IntClust subgroups were risk factors for visceral-only or bone-only first metastases. Among gene modules, those related to proliferation increased the risk of metasynchronous metastasis (OR (95% CI) = 2.3 (1.1–4.8)) and visceral-only first metastasis (OR (95% CI) = 2.5 (1.2–5.1)) but not bone-only metastasis (OR (95% CI) = 0.97 (0.56–1.7)). A 21-gene module (*BV*) was identified in estrogen-receptor-positive breast cancers with metasynchronous metastasis to bone and viscera (area under the curve = 0.77), and its expression increased the risk of bone and visceral metasynchronous spread in this population. *BV* was further orthogonally validated with NanoString nCounter in primary breast cancers, and was reproducible in their matched lymph nodes metastases and an external cohort.

**Conclusion:**

This case-control study of WG-DASL global expression profiles from FFPE tumour samples, after careful quality control and RNA selection, revealed that gene modules in the primary tumour have differing risks for clinical patterns of metasynchronous first metastases. Moreover, a novel gene module was identified as a putative risk factor for metasynchronous bone and visceral first metastatic spread, with potential implications for disease monitoring and treatment planning.

**Electronic supplementary material:**

The online version of this article (doi:10.1186/s13058-017-0881-y) contains supplementary material, which is available to authorized users.

## Background

Development of metastatic breast cancer is a complex multi-step process manifesting with diverse temporal patterns involving single or multiple organs [1]. Metastases to multiple bone or visceral sites may be recorded as synchronous (reported at the same time), metasynchronous (where reported metastases are separated by a short time period, typically months) or asynchronous events with a significant delay between distant recurrences [2–4]. The median survival of patients with metastatic breast cancer is 18 to 24 months, although the range in survival spans between a few months and many years and often depends on the pattern or burden of metastatic spread. Most clinicians recommend initial treatment with chemotherapy for rapidly progressive visceral disease or in women with severe symptoms related to metastatic breast cancer [5]. Patients with bone metastases are often treated with osteoclast inhibitors, as these agents have been shown to reduce the risk of skeletal related events such as fractures, the need for surgery or radiation to bone, spinal cord compression, and hypercalcaemia of malignancy. However, patients at risk of metasynchronous metastatic spread to bone and viscera may benefit from an alternative treatment strategy at the time of first metastatic presentation. Clinicians may pursue more aggressive therapy immediately (e.g. chemotherapy instead of endocrine therapy) in patients with bone metastasis who are at high likelihood of imminent visceral metastasis, or similarly add bone-directed therapy in patients with visceral metastasis who are at high likelihood of imminent bone metastasis. Identification of these patients at an early stage after primary diagnosis or during early metastatic disease is not well-established.

Various prognostic factors influence the overall survival of patients with metastatic breast cancer, including hormone receptor status and axillary lymph node status at diagnosis, previous adjuvant chemotherapy, and the number of involved organs [6, 7]. ER-positive disease has a predilection to metastasise to bone, whereas basal-like and claudin-low breast cancers are associated with brain and lung relapses as first site of metastasis and human epidermal growth factor receptor 2 (HER2)-positive tumours have a predilection to cerebellar metastasis [8–14]. Transcriptional features present in the primary invasive breast carcinoma can be intrinsic to metastatic progression [15] and are currently tested in clinical trials for patient stratification for treatment regimens [16, 17]. IntClust subtypes can stratify patients by disease-specific survival [18], but without attribution to specific metastatic patterns. Recently small-scale studies suggested circulating tumour-derived exosomes to be predictive of metastasis to individual bone or visceral metastatic sites [19]. It remains unclear, however, to what extent the primary tumour at the time of diagnosis confers risk factors for clinical patterns of disease progression, manifesting as diverse temporal patterns of metastatic spread to single or multiple different organ sites.

With the increasing use of small diagnostic biopsy procedures prior to systemic treatment in cancer patients, there are limited opportunities to also collect frozen tissue. The latter has been the prime resource for genomic analysis and subsequent publication of gene signatures for prognostic and predictive use. However, despite the potentially vast resource available within diagnostic formalin-fixed paraffin-embedded (FFPE) archives, they have remained largely untapped for exploratory genome-scale biomarker studies. The quality of RNA from FFPE material has been the key limitation to its subsequent use. Whilst modifications to the extraction techniques continue to make slight improvements to the degraded RNA, there have been greater developments in array-based gene expression profiling assays and emerging technologies for transcriptome sequencing from FFPE samples [20]. The Illumina Whole-Genome DASL (WG-DASL) assay is one such assay [21]. Several technical studies reported that DASL assays can produce reliable expression profiles from FFPE tumour tissue samples, given adequate RNA quality, and design and preprocessing of the resulting data [21–28].

Here, we designed a whole-genome expression profiling study using a case-control design to include primary invasive breast cancers with three clinically observed patterns of metastatic spread: (1) metasynchronous bone and visceral metastases (within 6 months of first metastasis); (2) bone only, with delayed or no visceral metastasis; and (3) viscera only, with delayed or no bone metastasis. Given the comprehensive time and detailed organ-site information in our cohort from which cases and controls were selected, we aimed to identify intrinsic molecular features of primary breast carcinomas associated with the distinct patterns of first metastatic spread observed in the clinic, and in particular those with metasynchronous bone and visceral metastases.

## Methods

### Study population, study design, and patient selection

The study population comprised 5061 patients diagnosed with invasive primary breast cancer without distant metastasis at the time of diagnosis between 1975 and 2005 from Guy’s Hospital, London UK. All patients had given consent for analysis of their tumour tissues. Median follow up was 11 years (time between entry and exit dates in the case-control design).

Three metastatic populations were defined according to the site and specificity of recurrence: (1) first recurrence to bone only, with no other metastatic site within 6 months (“bone-only”), (2) first recurrence to viscera (other organs) only, with no other metastatic site within 6 months (“visceral-only”), and (3) recurrence to bone and viscera within a period of 6 months (“bone and visceral”). Bone metastasis was defined as distant metastasis to bone or bone marrow, spinal cord compression, pathological fracture, or hypercalcaemia. Visceral metastasis included distant metastasis to lung, liver, brain, or ascites. In the study population, 1598/5061 (32%) developed distant metastasis: 413/1598 (26%) to bone as first site (bone-only), 747 (47%) to viscera as first site (visceral-only), and 438 (27%) to bone and viscera within a 6-month period (bone and visceral).

**Fig. 1:**
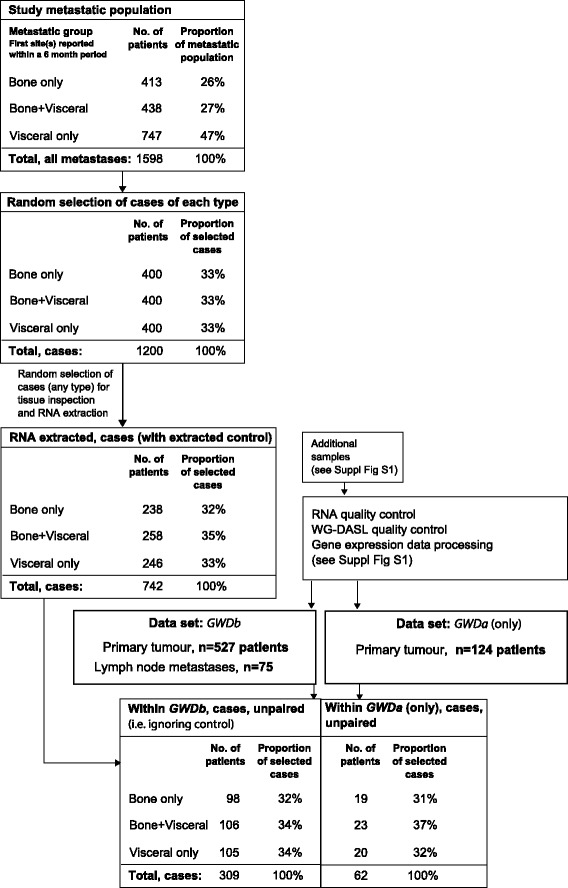
Overview of cases and gene expression datasets. For each metastatic population (Visceral only, Bone + Visceral, and Bone only) 400 cases were sampled, and three possible controls were matched to each case by calendar time of event. A random sample of case-control sets was taken forward to tissue assessment for RNA extraction (Additional file 1: Supplementary methods). Extracted RNA was available for a total of 742 case-control (1:1) pairs, comprising a total of 1277 individual patients. A detailed overview of patients and samples included in the design and in the gene expression datasets is shown in Additional file 2: Figure S1A, B

For each of the three metastatic populations (visceral-only, bone and visceral, and bone-only), individually matched controls were randomly sampled using a case-control incidence-based approach (Additional file 1: Supplementary methods). Briefly, for each calendar time, *T* (e.g. 15 January 1999) that a case is diagnosed, one or more controls are randomly selected from the other members of the cohort who, at time *T*, are still at risk of developing the outcome (distant metastasis). The controls are therefore matched to the case by time of event. A patient who is a control at one time can later become a case and/or a control again, and each of the control series therefore includes a combination of patients still at risk, which enables efficient estimation of risks in the clinical population (among 1200 case-control sets, 75% of patients selected as controls at any calendar time did not metastasize at all, and 25% went on to have a distant metastatic event). Case-control sets (n = 400) were randomly selected for each of the three metastatic populations, giving a total of 1200 selected case-control sets. Case-control sets were then selected for tissue assessment and RNA extraction from FFPE tissue blocks. Extracted RNA was available for a total of 742 case-control (1:1) pairs: 246 case-control pairs for visceral-only cases, 258 for bone and visceral cases, and 238 for bone-only cases. An overview of patient cases is shown in Fig. 1 and a detailed overview of case-control sampling, random selection, and extracted case-control pairs is shown in Additional file 2: Figure S1A and B. Additional file 3: Table S1 tabulates the number of cases with extracted case:control pairs (1:1), and the number of matched and unmatched cases and controls with available gene expression data.

### RNA extraction and gene expression profiling

FFPE samples of breast carcinomas were micro-dissected following tissue review. A total of 1575 FFPE tissue blocks were assessed (H&E; Additional file 1: Supplementary methods). Primary tumour blocks from 1357 patients were taken forward to micro-dissection and RNA extraction, with a total of 1370 RNA samples (Additional file 2: Figure S1A). In addition, RNA was extracted from 100 matched positive axillary lymph nodes and from FFPE samples of six breast cancer cell lines. RNA extraction was outsourced to Gen-Probe Life Sciences Ltd (Manchester, UK). RNA sample quality, quantity and integrity were assessed before proceeding to Illumina HT-12 v4 BeadChips WG-DASL microarray. A detailed description of tissue selection, micro-dissection, RNA sample selection, hybridisation design and microarray data processing is provided in Additional file 1: Supplementary methods. Two gene expression datasets were produced following rigorous quality assessment: GWDb (containing primary tumour samples from 527 patients) and GWDa (containing primary tumour samples from 124 patients, after removing patients also present in GWDb). Patient characteristics for GWDb are provided by case-control series in Table 2. An overview diagram of each dataset is provided in Additional file 2: Figure S1A. Gene expression microarray data have been deposited to Array Express E-MTAB-4003.

### Intrinsic subtype assignments and gene module scores

Prediction analysis of microarray 50 (PAM50) intrinsic subtype was assigned in accordance with Weigelt et al. [29] using median-centred data and matching probes to centroid identifiers by gene symbol. The nearest centroid identified by Spearman correlation was assigned to each sample. IntClust subtypes were assigned using the iC10 package (v1.1.2) [30] for R/Bioconductor. Gene module scores for a panel of previously reported gene modules were estimated using the Denoising Algorithm based on Relevance network Topology (DART) method [31] and further compared with weighted sum (weights (+1, −1) according to the direction of expression in the gene signature) (Additional file 1: Supplementary methods). Previously reported gene expression signatures were mapped to WG-DASL probes using Ensembl Gene ID, Entrez Gene ID or gene symbol, according to their original source (Additional file 3: Table S2). Where multiple microarray probes mapped to a single Entrez Gene ID, the probe with the most variable gene expression across the datasets was used (based on standard deviation in the relevant dataset).

### Derivation and expression summary of gene module

To identify a candidate gene module for bone and visceral metastasis (*BV* gene module), GWDb was reduced to ER-positive case-control pairs, and top-ranked genes were identified using an exploratory differential expression analysis of the bone and visceral and the no metastasis groups, as follows. Top-ranked genes were identified by comparing bone and visceral vs. no metastasis (threshold false discovery rate (FDR)-adjusted *p* < 0.2; Mann-Whitney *U* test). This procedure resulted in a list of 21 genes (19 up, 2 down) together with the direction of differential expression between the bone and visceral and the no metastasis groups (up, down) which defines the *BV* gene module. To inspect the candidate *BV* module within expression datasets, the expression of the gene module was summarised using a weighted sum with weights (+1, −1) according to the original direction of differential expression in the gene module.

### NanoString gene expression analysis

For a subset of 192 samples, expression was validated for 150 selected genes by analysing total RNA (200 ng) with the nCounter platform (NanoString Technologies). Expression data were normalised using the NanoStringNorm package in R [32]. Background correction was performed by subtracting the negative control probes (‘mean.2sd’). Expression values were normalised to the geometric mean of fifteen housekeeping genes. Expression values were log_2_-transformed and standardised within each sample (geometric mean). An expression score for the *BV* gene module was calculated among ER-positive samples using a weighted sum (weights (+1, −1) according to the direction in the *BV* module) of mean-centred, standard deviation-scaled *BV* genes.

### Statistical analysis

For each case-control series, conditional logistic regression models (modelling individually matched pairs) and logistic regression models (unconditional, disregarding the case-control matching) were used to estimate odds ratios (ORs) and 95% confidence intervals (CIs). For intrinsic molecular subtypes, ORs were estimated for each subtype compared with a baseline subtype. Immunohistochemically (IHC)-derived subtypes were compared with ER-positive, HER2-negative tumours [33]. PAM50 subtypes were compared with the luminal A subtype (a good prognosis group [34]). IntClust subtypes were compared with the baseline IntClust3 cluster [30]. Gene module scores were scaled within each case-control series so that 95% of values lay within the range [−1, 1] [35]. FDR/Benjamini-Hochberg correction for multiple testing was applied to *p* values across the panel of reported gene modules within each test [36]. A quartile analysis, in which cases were binned according to the quartile thresholds of the respective control series and conditional logistic regression models fitted for each quartile compared with the first quartile, showed similar trends in OR to the models that treat gene module scores as continuous variables (not shown). The Wilcoxon test for matched pairs and Mann-Whitney *U* test (unpaired) was used to test for differences in gene module scores between cases and controls in each series. All statistical analysis was conducted in the R environment (v3.1.2) (www.r-project.org). Conditional regression models were fitted using the function *clogistic()* in the package *Epi* (v2.0) [37]:

(Case_Control ~ x + strata(pair.id)).

Logistic regression models were fitted using the function *glm(family = ‘binomial’)* in the base package *stats*. A Sweave document is provided in Additional file 1: Supplementary methods.

## Results

### Patient characteristics and sample processing

Clinico-pathological information for extracted RNA samples from 742 cases and their case-matched controls are summarised in Table 1. A rigorous inspection of extracted RNA and WG-DASL data was performed to ensure that expression profiles could be obtained across the span of storage times and inferior-quality data were excluded from further analysis (Additional file 2: Figures S5-7). Primary tumour samples that passed rigorous WG-DASL quality controls were assigned to a discovery set (GWDb, 527 patients) or a smaller independent dataset (GWDa, 124 patients) (Additional file 2: Figure S1A, B). Clinico-pathological information for the three case-control series in dataset GWDb is shown in Table 2. Before proceeding to the analyses of the three case-control series, the clinico-pathological characteristics for each set were inspected. Patient characteristics in GWDb and GWDa retained the originally selected distribution of organ-specific metastatic spread (Additional file 3: Table S1A lists the number of cases for the case:control pairs (1:1), and the number of matched and unmatched cases and controls with available gene expression data). On inspection of GWDb, there were predominantly IHC-defined ER-positive breast cancers (~68%), and 18% IHC-defined HER2-positive and 32% IHC-defined ER-negative breast cancers. Primary carcinomas were predominantly treatment-naïve and invasive ductal carcinoma of no special histological type. Patients with a bone-only pattern of first metastatic spread were more likely to report a visceral metastasis beyond a 6-month period from first metastasis (37%) than the visceral-only group to a later bone metastasis (16%) (Additional file 3: Table S1B). The visceral-only case series had a greater proportion of grade-3 primary tumours and a smaller proportion receiving endocrine therapies than the other two case series in GWDb, while the bone-only case series had the lowest proportion of patients treated with chemotherapy (Table 2). Additional file 2: Figure S2 provides an illustration and descriptive summary of the temporal patterns of the single and multiple sites of metasynchronous metastatic spread present amongst all carcinomas in GWDb.

**Table 1:** Patient characteristics in the case-control series: patients with extracted RNA sample

		*V* cases	*V* controls	*BV* cases	*BV* controls	*B* cases	*B* controls
Number of patients		246	232	258	245	238	222
		Number (%)					
Age at hist. diagnosis	Median (years)	57.1	51.5	55.7	51.0	55.9	51.2
Grade	1	11 (4%)	33 (14%)	7 (3%)	36 (15%)	20 (8%)	20 (9%)
	2	81 (33%)	89 (38%)	113 (44%)	127 (52%)	114 (48%)	97 (44%)
	3	135 (55%)	87 (38%)	122 (47%)	62 (25%)	81 (34%)	88 (40%)
	Unknown	19 (8%)	23 (10%)	16 (6%)	20 (8%)	23 (10%)	17 (8%)
ER IHC status	Positive	147 (60%)	160 (69%)	162 (63%)	174 (71%)	188 (79%)	158 (71%)
	Negative	99 (40%)	72 (31%)	96 (37%)	71 (29%)	50 (21%)	64 (29%)
PR status	Positive	97 (39%)	121 (52%)	116 (45%)	131 (53%)	146 (61%)	113 (51%)
	Negative	149 (61%)	111 (48%)	142 (55%)	114 (47%)	92 (39%)	109 (49%)
HER2 status	Positive	58 (24%)	35 (15%)	55 (21%)	30 (12%)	35 (15%)	23 (10%)
	Negative	108 (44%)	102 (44%)	116 (45%)	98 (40%)	108 (45%)	107 (48%)
	Unknown	80 (33%)	95 (41%)	87 (34%)	117 (48%)	95 (40%)	92 (41%)
Tumour size	<= 2 cm	96 (40%)	113 (49%)	99 (38%)	136 (56%)	101 (42%)	117 (53%)
	>2 cm	146 (60%)	113 (49%)	155 (60%)	101 (41%)	133 (56%)	99 (45%)
	Unknown	-- --	6 (3%)	4 (2%)	8 (3%)	4 (2%)	6 (3%)
Lymph nodes positive	0	51 (21%)	97 (42%)	61 (24%)	109 (44%)	61 (26%)	103 (46%)
	1–3	74 (30%)	81 (35%)	64 (25%)	80 (33%)	70 (29%)	64 (29%)
	4+	74 (30%)	28 (12%)	81 (31%)	28 (11%)	64 (27%)	29 (13%)
	Unknown	47 (19%)	26 (11%)	52 (20%)	28 (11%)	43 (18%)	26 (12%)
Invasive subtype	NOS/no special type	217 (88%)	189 (81%)	219 (85%)	206 (84%)	196 (82%)	189 (85%)
	Ductal - mucinous	1 (0%)	3 (1%)	4 (2%)	2 (1%)	1 (0%)	4 (2%)
	Ductal - tubular	-- --	6 (3%)	1 (0%)	7 (3%)	3 (1%)	3 (1%)
	Ductal - other	6 (2%)	3 (1%)	1 (0%)	3 (1%)	1 (0%)	3 (1%)
	Lobular - classical	14 (6%)	23 (10%)	19 (7%)	22 (9%)	29 (12%)	13 (6%)
	Lobular - pleomorphic	3 (1%)	2 (1%)	6 (2%)	3 (1%)	3 (1%)	4 (2%)
	Lobular - other	2 (1%)	3 (1%)	3 (1%)	2 (1%)	4 (2%)	1 (0%)
	Other	3 (1%)	3 (1%)	5 (2%)	-- --	1 (0%)	5 (2%)
Surgery type (any time)	Breast conserving	58 (24%)	80 (34%)	70 (27%)	82 (33%)	64 (27%)	72 (32%)
	Breast conserving + mastectomy	53 (22%)	28 (12%)	56 (22%)	32 (13%)	37 (16%)	27 (12%)
	Mastectomy	106 (43%)	110 (47%)	99 (38%)	120 (49%)	115 (48%)	114 (51%)
	Unknown	29 (12%)	14 (6%)	33 (13%)	11 (4%)	22 (9%)	9 (4%)
Radiotherapy (adj/neo)	Yes	168 (68%)	140 (60%)	197 (76%)	126 (51%)	194 (82%)	125 (56%)
	No	78 (32%)	92 (40%)	61 (24%)	119 (49%)	44 (18%)	97 (44%)
Hormone treatment (adj/neo)	Yes	204 (83%)	120 (52%)	230 (89%)	130 (53%)	218 (92%)	114 (51%)
	No	42 (17%)	112 (48%)	28 (11%)	115 (47%)	20 (8%)	108 (49%)
Chemotherapy	Neo-adj only	-- --	3 (1%)	3 (1%)	67 (27%)	8 (3%)	3 (1%)
	Neo-adj and adj	6 (2%)	1 (0%)	9 (3%)	1 (0%)	1 (0%)	2 (1%)
	Adj only	146 (59%)	85 (37%)	150 (58%)	2 (1%)	101 (42%)	67 (30%)
	No	94 (38%)	143 (62%)	96 (37%)	175 (71%)	128 (54%)	150 (68%)

*Hist*. histological assessment, *ER* IHC estrogen receptor status determined by immunohistochemical assessment, *PR* progesterone receptor, *HER2* human epidermal growth factor receptor 2, *adj* adjuvant, *neo* neoadjuvant

**Table 2:** Patient characteristics in the case-control series: patients in the discovery cohort *GWDb*

		*V* cases	*V* controls	*BV* cases	*BV* controls	*B* cases	*B* controls	Case series	
Number of patients		105	82	106	86	98	90	*χ* ^*2*^	*p*
Age at hist. diag.	Median (years)	55.4	52.0	52.5	50.3	55.9	48.8		
Grade	1	3 (3%)	12 (15%)	3 (3%)	13 (15%)	8 (8%)	7 (8%)		
	2	27 (26%)	32 (39%)	44 (42%)	47 (55%)	44 (45%)	42 (47%)		
	3	68 (65%)	34 (41%)	55 (52%)	19 (22%)	38 (39%)	37 (41%)		
	Unknown	7 (7%)	4 (5%)	4 (4%)	7 (8%)	8 (8%)	4 (4%)	18.2	0.01
ER IHC status	Positive	57 (54%)	52 (63%)	69 (65%)	62 (72%)	77 (79%)	65 (72%)		
	Negative	48 (46%)	30 (37%)	37 (35%)	24 (28%)	21 (21%)	25 (28%)	13.3	0.001
PR status	Positive	39 (37%)	40 (49%)	53 (50%)	56 (65%)	59 (60%)	51 (57%)		
	Negative	66 (63%)	42 (51%)	53 (50%)	30 (35%)	39 (40%)	39 (43%)	10.9	0.004
HER2 status	Positive	31 (30%)	13 (16%)	22 (21%)	13 (15%)	15 (15%)	11 (12%)		
	Negative	44 (42%)	38 (46%)	51 (48%)	41 (48%)	45 (46%)	42 (47%)		
	Unknown	30 (29%)	31 (38%)	33 (31%)	32 (37%)	38 (39%)	37 (41%)	7.0	0.1
Tumour size	<= 2 cm	41 (39%)	39 (48%)	39 (37%)	44 (51%)	38 (39%)	47 (52%)		
	>2 cm	64 (61%)	42 (51%)	65 (61%)	38 (44%)	60 (61%)	41 (46%)	0.1	1.0
	Unknown	-- --	1 (1%)	2 (2%)	4 (5%)	-- --	2 (2%)		
Lymph nodes positive	0	15 (14%)	33 (40%)	21 (20%)	32 (37%)	24 (24%)	39 (43%)		
	1–3	34 (32%)	29 (35%)	31 (29%)	28 (33%)	30 (31%)	30 (33%)		
	4+	37 (35%)	10 (12%)	40 (38%)	11 (13%)	26 (27%)	10 (11%)		
	Unknown	19 (18%)	10 (12%)	14 (13%)	15 (17%)	18 (18%)	11 (12%)	6.1	0.4
Invasive subtype	NOS/no special type	96 (91%)	70 (85%)	85 (82%)	73 (85%)	82 (84%)	81 (90%)		
	Ductal - mucinous	-- --	2 (2%)	1 (1%)	-- --	1 (1%)	1 (1%)		
	Ductal - tubular	-- --	-- --	1 (1%)	3 (3%)	2 (2%)	1 (1%)		
	Ductal - other	1 (1%)	1 (1%)	1 (1%)	-- --	-- --	3 (3%)		
	Lobular - classical	5 (5%)	5 (6%)	7 (7%)	9 (10%)	12 (12%)	2 (2%)		
	Lobular - pleomorphic	1 (1%)	1 (1%)	6 (6%)	-- --	-- --	1 (1%)		
	Lobular - other	-- --	2 (2%)	2 (2%)	1 (1%)	1 (1%)	-- --		
	Other	2 (2%)	1 (1%)	1 (1%)	-- --	-- --	1 (1%)	1.7	0.4 †
Surgery type (any time)	Breast conserving	30 (29%)	34 (41%)	35 (33%)	43 (50%)	31 (32%)	39 (43%)		
	Breast conserving + mastectomy	17 (16%)	11 (13%)	19 (18%)	7 (8%)	15 (15%)	13 (14%)		
	Mastectomy	47 (45%)	33 (40%)	41 (39%)	30 (35%)	43 (44%)	35 (39%)		
	Unknown	11 (10%)	4 (5%)	11 (10%)	6 (7%)	9 (9%)	3 (3%)	1.2	1.0
Radiotherapy (adj/neo)	Yes	79 (75%)	52 (63%)	84 (79%)	55 (64%)	82 (84%)	59 (66%)		
	No	26 (25%)	30 (37%)	22 (21%)	31 (36%)	16 (16%)	31 (34%)	2.2	0.3
Hormone treatment (adj/neo)	Yes	84 (80%)	38 (46%)	97 (92%)	57 (66%)	92 (94%)	51 (57%)		
	No	21 (20%)	44 (54%)	9 (8%)	29 (34%)	6 (6%)	39 (43%)	11.0	0.004
Chemotherapy	Neo-adj only	-- --	2 (2%)	2 (2%)	1 (1%)	5 (5%)	3 (3%)		
	Neo-adj and adj	4 (4%)	-- --	4 (4%)	1 (1%)	1 (1%)	-- --		
	Adj only	65 (62%)	33 (40%)	67 (63%)	24 (28%)	43 (44%)	28 (31%)		
	No	36 (34%)	47 (57%)	33 (31%)	60 (70%)	49 (50%)	59 (66%)	8.7	0.01 ‡
Metastatic events in control series		No mets, 64 (78%) V = 8, BV = 8, B = 2		No mets, 66 (77%) V = 10, BV = 5, B = 5		No mets, 69 (77%) V = 4, BV = 10, B = 7			

†ductal versus lobular, ‡any versus no chemotherapy. Abbreviations as for Table 1

### Metastatic spread among breast cancer subtypes

Next we asked to what extent patients with particular molecular subtypes of breast cancer, as currently defined in the research setting, were at risk of metasynchronous bone and visceral, bone-only, or visceral-only patterns of first metastasis observed in the clinic. Molecular subtypes in the GWDb set were defined by the IHC-defined status of ER/HER2 [33], and assigned to the PAM50 [34] and the IntClust subtypes [18, 30]. Initially, PAM50 estimates for each tumour were compared with IHC-defined subtypes and overall good accordance was observed: luminal A samples were 89% IHC-defined ER-positive; 62% of basal-like samples were of the triple-negative phenotype (IHC-defined ER-negative, PgR-negative, and HER2-negative; TNBC); and 73% of HER2-enriched samples were IHC-defined HER2-positive among samples with available IHC status.

Second, the molecular subtypes were tested within each case-control series using conditional logistic regression (Additional file 4: Table 3). IHC-defined ER-positive patients had increased risk of bone-only metastatic spread and decreased risk of visceral-only and bone and visceral metastatic spread. In GWDb, patients with the HER2-enriched PAM50 subtype of breast cancer had increased risk of visceral-only metastatic spread, and patients with the luminal B subtype had increased risk of bone-only metastatic spread, compared with patients with the luminal A subtype as baseline. Patients with tumours classified as IntClust5 had an increased risk of visceral-only spread compared to those with IntClust3 baseline tumours. Unconditional logistic regression models had similar OR point estimates (Additional file 5: Table 4). Patients with the IHC-defined ER-negative/HER2-positive subtype had an increased risk of visceral-only and bone and visceral spread compared to those with ER-positive/HER2-negative carcinomas. Among IntClust classes with IntClust3 as the reference class, patients with bone and visceral spread had similar risks to the visceral-only group with the exception that IntClust2 and IntClust4 showed increased risk for visceral-only and bone-only but not bone and visceral spread. Subtypes found to be risk factors for bone and visceral spread were also risk factors for visceral-only or bone-only events from either the conditional or unconditional logistic regression models, indicating that molecular subtypes did not confer risks specifically for bone and visceral events in this study.

**Fig. 2:**
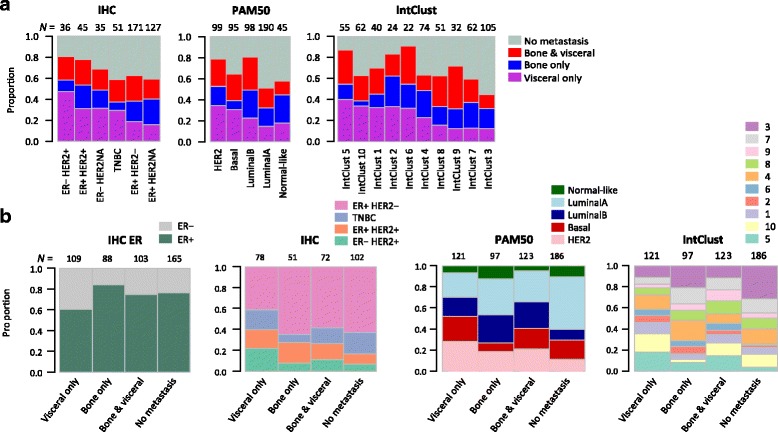
Distribution of three metastatic groups across breast cancer subtypes within the dataset GWDb. Barplots illustrate the proportion of immunohistochemically defined (IHC), prediction analysis of microarray 50 (PAM50) and IntClust breast cancer subtypes present in each metastatic group (**a**) and the proportion of each metastatic group assigned to IHC, PAM50 and IntClust subtypes (**b**). The patient number for each group is shown on the top of each column. ER estrogen receptor, HER2 human epidermal growth factor receptor, TNBC triple-negative breast cancer

Third, the tumour molecular subtypes of all patients in GWDb were tabulated and compared to the metastatic pattern of every patient irrespective of the case-control design, with the aim of providing a descriptive overview [30] of all primary tumours in GWDb (Fig. 2). As predicted, IHC-defined ER-negative and HER2-positive tumours were enriched for the visceral-only cases, and TNBC was decreased for bone-only events (Fig. 2, IHC). The breakdown of IHC subtypes in the bone and visceral group lies in between the bone-only and visceral-only groups and does not appear to be dominated by the IHC associations that would be expected for one case type or the other (bone only or visceral only). The differences were less clear across the PAM50 subgroups (Fig. 2, PAM50). IntClust5 had the highest prevalence of visceral-only events, whereas IntClust9 followed by IntClust6 had the most bone and visceral events (Fig. 2, IntClust). The patients in the bone-only group had mainly ER-positive breast cancers and were predominantly assigned to IntClust3, 4 and 7 subtypes. In contrast, patients with no reported metastases were enriched for luminal A and IntClust3 subtypes (IntClust in the “no metastasis” group; χ^2^, *p* < 1e-5), and bone and visceral events were present in all IntClust subtypes (IntClust in the bone and visceral group; χ^2^, *p* = 0.3). In summary, primary breast cancers with bone and visceral metasynchronous metastatic pattern were found across multiple molecular breast cancer subtypes in GWDb, indicating that there was no evidence of increased risk specifically for bone and visceral events among these current breast cancer classifications (Additional file 4: Table 3 and Additional file 5: Table 4).

### Prognostic gene modules are indicative of organ-specific metastatic predilection

Several studies have reported gene expression modules indicative of particular organ-specific metastatic spread (reviewed in [38, 39]). We therefore asked whether some of those modules were also activated across our three metastatic groups and to what extent the activation of individual gene modules in the primary tumour is a risk for the clinically observed patterns of metasynchronous bone and visceral, bone-only, or visceral-only first metastasis. Primary tumour expression modules were selected if they were previously reported to be associated with: (i) features of proliferation, cell motility, presence of stem-cell-like cells and immune/lymphocytic infiltration, and (ii) organ-specific metastasis (Additional file 3: Table S2).

**Fig. 3:**
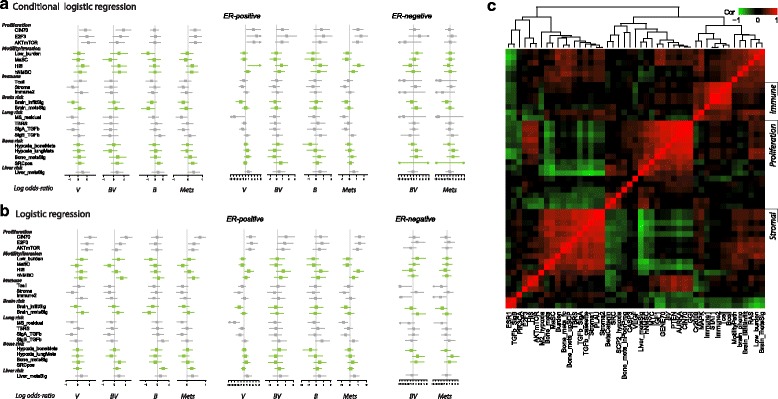
Log (OR) estimated from univariate conditional logistic regression and logistic regression models of illustrative gene modules in the GWDb dataset for each metastatic group. **a** Conditional logistic regression (matched pairs). **b** Logistic regression using complete case-control pairs. Alternating grey and green bars indicate the broad categorisation of illustrative gene modules (“Proliferation”, “Immune”, etc.) as shown in the module labels on the left of the plot. **c** Heatmap of pairwise correlation for a panel of gene modules (Pearson correlation, “complete” clustering; irrespective of case type or case-control status). Gene modules are listed in Additional file 3: Table S2. *ER*, estrogen receptor; *V*, "visceral-only"; *BV*, "bone and visceral"; *B*, "bone-only"; *Mets*, distant metastasis at any site

The gene modules were each tested as a risk for each pattern of metastatic spread within GWDb using conditional logistic regression (Fig. 3a) and logistic regression on complete case-control pairs (Fig. 3b). In addition, logistic regression models were fitted using all controls and all cases (to avoid discarding both samples from a pair due to missing data from GWDb), and using ER-stratified data with or without discarding samples from ER-mismatching pairs (Additional file 2: Figure S3). Pairwise correlation of gene modules confirms that proliferation signatures are highly correlated in this dataset and there is also correlation between other modules previously reported to represent metastasis to individual sites and between immune-related signatures (Fig. 3c), consistent with other studies [35, 40]. The expression of genes associated with proliferation has been repeatedly shown to be associated with the prognosis in ER-positive breast cancer [41]. In our study, gene modules related to proliferation increased the risk of metasynchronous bone and visceral metastasis (e.g. in the conditional logistic regression model of *PTEN* module*,* OR (95% CI) = 2.3 (1.1–4.8); Additional file 6: Table S3) and visceral-only first metastasis (*PTEN,* OR (95% CI) = 2.5 (1.2–5.1); Additional file 6: Table S3) but not bone-only metastasis (*PTEN*, OR (95% CI) = 0.97 (0.56–1.7); Additional file 6: Table S3). Risk associations were observed by logistic regression modelling within ER-positive or ER-negative tumours (Additional file 6: Table S3, Additional file 2: Figure S3A).

In addition, with the alternative aim of providing a descriptive summary of gene module activation among all breast carcinomas present in GWDb, two exploratory analyses were performed irrespective of the case-control design: tumours with each pattern of metastatic spread were compared with all tumours with no metastasis using a logistic regression model (Additional file 2: Figure S3B), and time to metastasis to individual sites (lung, liver, bone, brain) was modelled irrespective of the patterns of first metastatic spread or any other metastases at any time during follow up (Additional file 2: Figure S3C, D). In agreement with other studies, neither the logistic regression with reference to tumours with no metastasis nor the time-to-event analyses can be interpreted in the standard epidemiological sense estimating associations between exposures and outcomes, due to the sample selection methods employed in this study. These models are presented here as exploratory hypothesis-generating tools only with no inference implied for the breast cancer population. Metastasis to any site was associated with proliferation signatures irrespective of ER status (Additional file 2: Figure S3C). A number of gene modules indicated nominal significance but would not pass a multiple testing correction (Additional file 2: Figure S3). In IHC-defined ER-negative breast cancers transforming growth factor (TGF)-β response [42] and hypoxia response gene sets [43] were activated in carcinomas with visceral-only metastases compared with those that did not metastasise (Additional file 2: Figure S3B), while TGF-β response and hypoxia response gene sets were associated with time to lung metastasis (Additional file 2: Figure S3D). A stem cell module, which is a strong indicator of short relapse in TNBC [44], was present in ER-negative breast cancers with visceral-only metastases, and a module related to intermediate tissue burden and progression from stemness/basal-like cells [45] was associated with the visceral-only and bone and visceral groups, while the low tissue-burden/basal-like module (derived from metastatic cells from tissues with low metastatic burden [45]) was under-expressed in the bone-only group compared with cancers with no reported metastases. Taken together, while we were able to recapitulate previously reported associations between gene signatures and metastasis to bone or visceral organs within our study, there were no specific gene module with distinctive risk factors for the group with clinically observed metasynchronous bone and visceral spread.

### A prognostic gene module for metasynchronous metastatic spread

**Fig. 4:**
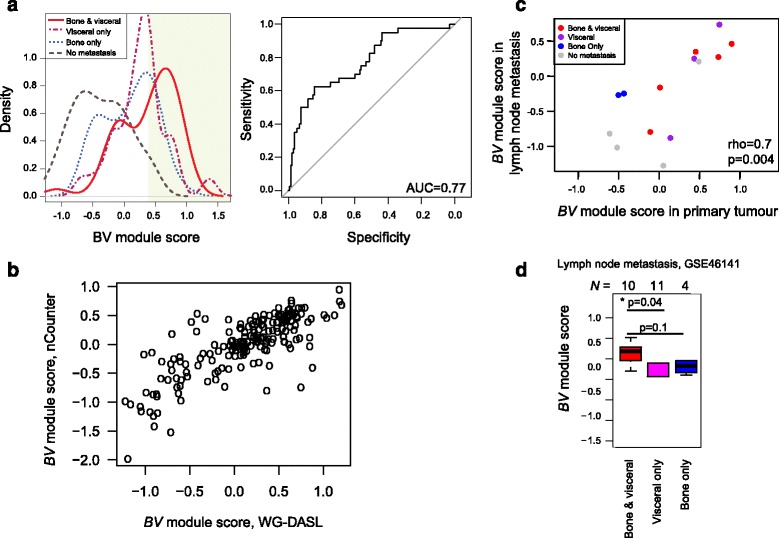
*BV* module for metasynchronous metastatic spread. **a** Discovery of the *BV* module. *BV* module scores, shown as density plots for each metastatic group in the estrogen-receptor-positive case-control paired breast cancer cases from the discovery set (GWDb) together with the corresponding receiver operating characteristic curve for the bone and visceral group. **b** Estimated *BV* expression score based on NanoString nCounter quantification compared with those obtained from the WG-DASL platform. **c** Correlation plot displaying the BV gene set in primary tumours and their matched lymph node metastases. Points are colour-coded according to metastatic group. **d** *BV* module scores in lymph node metastases of the GSE46141 data, displayed according to their reported patterns of first metastatic spread

As the question remains whether any molecular features could be identified in primary tumours at the time of diagnosis for metasynchronous bone and visceral metastases, we then aimed to extract specific gene expression patterns associated with the bone and visceral metastasis group. In order to control for interactions between ER status and metastatic group, the discovery set (GWDb) was stratified by ER status by removing those case-control pairs with differing IHC-defined ER status (Additional file 1: Supplementary methods). We proceeded with a total of 175 ER-positive breast cancer patients. Exploratory differential expression analysis was performed between the bone and visceral group and tumours that did not metastasise, and the top-ranked genes (FDR-adjusted *p* < 0.2; see “Methods”) were taken forward for further exploratory analysis (Additional file 3: Table S4). In receiver operating characteristic (ROC) curve analysis the area under the curve (AUC) was 0.77 for a 21-gene set for the bone and visceral metastasis group (“*BV* module”; Fig. 4a, Additional file 3: Table S4), in comparison to an AUC of 0.66 and 0.56 for visceral-only and bone-only metastatic spread, respectively, while when combining all three series, the overall AUC was 0.83 for any metastatic site (Additional file 2: Figure S4).

The risk of bone and visceral spread from the *BV* gene module was next estimated using the bone and visceral case-control series within GWDb and GWDa (Additional file 3: Table S5). A significant risk of bone and visceral spread was observed within GWDb (OR (95% CI) = 6.0 (3.1–12.2). In the independent dataset GWDa, the OR estimates were also positive (OR (95% CI) = 1.9 (0.38–9.7), and there was a shift towards increased *BV* module scores in the cases compared to the controls (Mann-Whitney *U* test*, p* = 0.3), however due to the small sample size it was not significant (Additional file 3: Table S5). Together these results indicated that this *BV* gene module might confer an increased risk of the bone and visceral pattern of metastatic spread.

To further explore the relevance of our *BV* gene module, orthogonal validation of the discovery was obtained on three levels: (i) with NanoString nCounter, by testing the expression of these genes in a representative subset of 192 samples, in which *BV* module scores were highly concordant with WG-DASL values (Fig. 4b; Pearson’s correlation = 0.78, *p* < 1e-4); (ii) the expression of the *BV* gene set was reproduced in matched lymph node metastases within our cohort (Fig. 4c); and (iii) we investigated the gene expression of the *BV* module in an external dataset of lymph node metastases from breast cancers with known metastatic disease [46]. The *BV* gene module exhibited increased expression in the lymph node metastases of those patients with a bone and visceral pattern of first metastatic spread compared with visceral-only (Fig. 4d; Mann-Whitney *U* test, *p* = 0.04) and bone-only groups (Fig. 4d; Mann-Whitney *U* test, *p* = 0.1 (not significant)). These results are in line with our exploratory analyses and together are the first demonstration towards developing an intrinsic risk factor in primary breast cancer for metasynchronous bone and visceral first distant recurrence. Inspection of the genes comprising the candidate *BV* module indicated enrichment for association with condensing chromosomes and the kinetochore (Additional file 2: Figure S8).

## Discussion

Metastasis represents the major cause of death in breast cancer patients. Over the last few years, numerous molecular-based prognostic tests of varying specificity have emerged, indicating that primary breast carcinomas display expression profiles associated with organ-specific dissemination; however, few studies have addressed synchronous and metasynchronous patterns of metastatic spread [47]. Treatment strategies and monitoring of patients could potentially be tailored if prediction of single or multi-organ metastasis could be estimated at an early stage. As a step towards this goal, this study estimated potential risks for particular patterns of metastatic spread associated with intrinsic subtypes and gene modules in the primary tumour. A gene expression module present in primary invasive carcinomas associated with concurrent or short-term delays between the development of bone and visceral metastasis was identified and validated in an independent series of lymph node metastases.

Predilection for metastatic spread in breast cancer has previously been associated with gene modules enriched in the primary tumour. A common feature of these are markers of cell proliferation, such as the GENE70, PTEN, the centrosomal kinase *AURKA* [48, 49] and multiple processes related to chromosomal instability including CIN70 [50]. In this study, we found that a gene module containing components of the kinetochore (*CENPO, SPC25, CASC5, SKA3, CENPE*; Gene Ontology (GO) CC term “kinetochore”) was associated with the occurrence of metasynchronous bone and visceral metastases within 6 months of the first metastasis. The regulation of genes encoding kinetochore components has been hypothesised to drive chromosomal instability [51], whereby the upregulation of kinetochore genes may reflect the activation of a cell division programme [52]. We speculate that the association of this gene module with rapid multitropic bone and visceral spread after first metastasis points to a mechanism of chromosomal instability, enabling the development of subclones and selection of metastatic tumour cells for invasion and adaptation at multiple bone and visceral sites. Gene modules related to proliferation or mammary stem cells might be expected to influence the synchronicity of multiple metastases, and were indeed found to be significant risks for multiple bone and visceral first metastases.

Limitations of this study include the imposition of a timeline that defines metasynchronous metastases: for example, in our datasets a change in the definition of metasynchronous from 6 months to 12 months would have led to 10% of bone-only and 5% of visceral-only metastases to have been considered metasynchronous (Additional file 2: Figure S2A), and other definitions of metasynchronicity could be imposed, which may affect the estimated risks for each case type. The AUC for the *BV* signature was higher for all metastases than for metasynchronous bone and visceral metastases: from the point of view of clinical translation, further work would need to establish whether *BV* or other putative signatures for patterns of metastatic spread could add value over existing signatures such as Oncotype or Mammaprint [16, 17].

Metastasis is a complicated, multi-step process and our understanding of the multiple factors involved is still partial. In the last decade, genomic profiling has attempted to fill this knowledge gap; however, these studies have primarily used fresh-frozen tissue, had restricted numbers of primary and metastatic cases, and incomplete information on the site and time to development of the metastatic spread. This has limited the utility and clinical applicability of these modules. There is evidence that some tumours have a predilection for colonising specific tissues in clinical populations (e.g. [10]), while animal model and recent next-generation sequencing studies also support a role for subclonal adaptations to the metastatic niche (e.g. [53]). In this study, we focused on the tumour as one part of this complex metastatic cascade, which is close to clinical diagnostic practise and patient management. We hypothesised that intrinsic subtypes and gene modules confer risk of particular patterns of metastatic spread in some tumours. We addressed this question by designing a case-control study for particular patterns of metastatic spread.

Pre-clinical models have contributed to our understanding of metastastic spread but they might not capture many of the processes that are important in a clinical setting - including alterations to the immune system or incorporation of specific latency periods to study multi-organ metastatic spread in parallel - and many gene signatures originate from ER-negative cell line and patient-derived xenograft (PDX) models. Multiple lines of evidence indicate that intrinsic subtypes and gene modules have different metastatic potential within clinical populations [18, 54, 55]. We sought to address whether gene modules could confer risk for specific temporal patterns of metastatic spread using a large tumour archive with detailed clinical follow up. An efficient case-control design is required given that multiple breast cancer subtypes and metastatic patterns are present in any clinical population. This study therefore focused on three specific patterns of metastatic spread based on epidemiological observations from the same clinical population [56].

As recently shown by Iddawela et al*.* [28] and others, the WG-DASL platform together with stringent quality control and data processing steps can produce reliable results from FFPE breast tumour tissue. Here, by starting with a well-characterised cohort of 5061 patients with long-term follow up of whom 1598 developed metastasis, we have created a well-annotated and sufficiently large cohort to investigate molecular risk factors for single and multiple organotropic metasynchronous metastatic disease. While many samples were excluded during the quality control steps, the processed gene expression datasets enabled an investigation of gene expression modules as risk factors for specific patterns of first metastatic spread.

The use of an incidence-based case-control design ensured that non-metastasising primary tumour samples were also included from the same range of calendar dates of diagnosis, and enabled efficient estimation of effects in a standard clinical population. Cunha et al*.* [57] recently reported using a case-control design to estimate the effect of *ALK1* expression in frozen breast tumours as a molecular risk factor for metastatic spread. Here, our use of an incidence-based case-control design enabled the estimation of genome-wide molecular risk factors for three clinically observed patterns of first metastatic spread (bone-only, visceral-only, or bone and visceral within a 6-month time period). Further work on patterns of long-term disease progression may focus on a more defined population, such as ER-positive/HER2-negative or high-grade tumours, and make use of appropriate platforms for assaying fewer FFPE tumour samples from a stratified clinical population.

Patients at risk of metasynchronous bony and visceral metastases could potentially profit from closer disease monitoring and may benefit from a more radical bisphosphonate and chemotherapeutic combination treatment strategy up front in the metastatic setting. In ER-positive disease the benefits of hormonal therapy in managing visceral metastases from breast cancer are much lower than those offered by chemotherapy. Conversely, Perez et al*.* [58] suggest that, in patients with bone metastases, efforts should be made to select the least aggressive therapy to avoid excessive toxicity*.* Progress towards the optimisation of disease monitoring and treatment strategies fundamentally requires a better understanding of risks that could be estimated at primary diagnosis, together with an improved understanding of additional emerging risks as breast cancers progress to a metastatic setting (for example, establishment of a metastatic niche). Initiatives such as AURORA, a large multinational, collaborative metastatic breast cancer molecular screening programme [59] will further shed light on our knowledge of whether the gene expression patterns found in primary breast cancer is similar to those in metastatic material. This would add to our understanding of the metastatic process and guide treatment regimens for metastatic breast cancer. A liver-selective gene module was among the set of gene modules, where these genes were under-expressed in primary tumours from patients who subsequently developed liver metastases, but displayed high expression in the liver metastases [46]. We observed the low-level expression of these 17 genes in ER-positive cancers, which metastasised to the liver. Of note, this inverse correlation in the direction of transcript levels of some genes between primary tumours and metastases was not unique to this gene module, but was also seen in another gene module associated with infiltration of the blood-brain barrier [60] and has also been recently reported in ovarian cancers [61]. Due to the scarcity of available samples, a clear biological conclusion cannot be drawn about such inverse correlation and we hypothesise that the process of adaption to the new microenvironment or development of the pre-metastatic niche [62, 63] favours those primary tumour traits. Ultimately, larger cohorts with matched primary and metastatic lesions are required to elucidate the clinical relevance of these transcriptional changes after primary diagnosis.

## Conclusions

In conclusion, by analysing gene expression profiles in a large cohort of well-characterised primary breast carcinomas and lymph node metastases in patients with long-term follow up and detailed information on metastatic spread, we were able to identify patterns informative of multiple organotropic metasynchronous metastatic spread. Further investigations are necessary in order to tease out the contributing components, which could be relevant for tailoring systemic therapeutic regimens, monitoring response or resistance to therapy, and warranting close imaging/biomarker follow up with the institution of early intervention strategies as required. These genome-wide expression data across extensive case-control series will provide a useful resource facilitating further studies of the biology and clinical relevance of single and metasynchronous metastatic spread, and might enable rational personalised treatment strategies to be developed for patients at risk of bone or visceral metastasis with subsequent metasynchronous metastasis.

## Additional files

Additional file 1: Supplementary methods. Detailed description of tissue preparation including microtomy, de-waxing and staining, micro-dissection and preparation of materials, and gene expression processing and quality control. (PDF 243 kb)

Additional file 2: Figures S1-S8.. (PDF 2279 kb)

Additional file 3: Tables S1-S2 and S4-S5. (XLSX 365 kb)

Additional file 4: Table 3. Estimated ORs for molecular subtype representation based on conditional logistic regression. (PDF 1 mb)

Additional file 5: Table 4. Estimated ORs for molecular subtype representations based on logistic regression. (PDF 657 kb)

Additional file 6: Table S3. Distribution of gene module scores for cases and controls in each case-control series, and estimated ORs using conditional logistic and logistic regression models. (XLSX 47 kb)

## Abbreviations

AUC: Area under the curve
BH: Benjamini-Hochberg method for multiple testing correction
ER: Estrogen receptor
FDR: False discovery rate
FFPE: Formalin-fixed, paraffin-embedded
HER2: Human epidermal growth factor receptor 2
IHC: Immunohistochemistry
PAM50: Prediction analysis of microarray 50
PgR: Progesterone receptor
TNBC: Triple-negative breast cancer
