# Supplementary material for: Gene expression modules in primary breast cancers as risk factors for organotropic patterns of first metastatic spread: a case control study
Source: Breast Cancer Res. 2017 Oct 13;19:113. doi: 10.1186/s13058-017-0881-y (PMC5640935; doi:10.1186/s13058-017-0881-y)
Supplement: Supplementary file 1 — Supplementary methods. Detailed description of tissue preparation including microtomy, de-waxing and staining, micro-dissection and preparation of materials, and gene expression processing and quality control. (PDF 243 kb) [file 13058_2017_881_MOESM1_ESM.pdf]

Supplementary Information, Lawler *et al.* (2017)

CONTENTS

Supplementary Methods
Supplementary References
SWEAVE document

SUPPLEMENTARY METHODS

Study design and patient selection

The sampling procedure was based on a case-control incidence density, or incidence based sampling. In this procedure the selection of controls is governed by the diagnoses of cases. Each calendar time, T (e.g. 15th January 1999) that a case is diagnosed, one or more controls are randomly selected from the other members of the cohort who, at the time T, are still at risk of developing the outcome (distant metastasis). The controls are therefore matched to the case by time of event. A patient who is a control at one time can later become a case and/or a control again.

For each metastatic population ("*visceral only*", "*bone & visceral*", "*bone only*") 400 cases were sampled, and three possible controls were matched to each case by calender time of event. This procedure defined 400 case-control sets for each of the three metastatic populations, giving a total of 1,200 case-control sets. A random sample of case-control sets was taken forward to tissue assessment for RNA extraction: the case tissue block was reviewed, and control tissue blocks were reviewed in turn until a control tissue block was identified for RNA extraction. Extracted RNA was available for a total of 742 case-control (1:1) pairs, comprising a total of 1,277 individual patients. Extensive clinico-pathological features, site and date of diagnosis of metastastic spread, as well as breast-cancer specific death data was available.

Cell lines

FFPE samples from six breast cancer cell lines (MDAMB231, HCC1954, HCC1143, T47D, MCF7, BT474) were sent for RNA extraction to Gen-Probe Life Sciences Ltd (UK) and assayed in technical duplicate. Cell line samples from a given duplicate pair were assayed on different hybridisation plates and preprocessed as part of data set *GWDb* as part of the quality control assessment procedure.

Tissue preparation

FFPE tumour blocks from the study cohort were obtained from the archive and an HE stained section was prepared for pathological review. Tissue samples underwent a standardised selection and microdissection protocol.

Microtomy

All surfaces, including microtome, surrounding bench, forceps, glass trough and brushes, were thoroughly cleaned with a paraffin cleaner (J.T. Baker Paraffin Cleaner cat. no. 3451) and then RNase Zap (Ambion cat. no. AM9780) to ensure sections will be cut in an RNase-free environment. The cleaned glass trough was placed in a water bath half-filled with deionised water and pre-heated to 40^0C. The trough was then filled with warm DEPC-treated water to create a bath within a bath in which the sections could be floated, this reducing the amount of DEPC water used. The DEPC-treated water was changed every two cases or more often if necessary. All other surfaces and instruments were thoroughly cleaned as described above between each case and a fresh blade, also cleaned with RNase Zap, used to cut each case. Depending on the surface area of the invasive carcinoma and cellularity, either four (invasive tumour >200mm^2 with >50% cellularity), six (invasive tumour between 200mm^2 with <50% cellularity and 100mm2 with >50% cellularity) or eight (invasive tumour >100mm^2 with <50% cellularity) sections with a thickness of 8μm were cut from each case and collected on sterilised slides. Sections were then either baked for 2 hours at 60^0C or stored at 4^0C overnight and then baked the following day prior to de-waxing. Sections were stored for no more than 24 hours before micro-dissection.

De-waxing and staining

All containers and instruments were sterilised prior to use by exposing to UV light for a minimum of 20 min before beginning the de-waxing process. Slides were immersed in Coplin jars of xylene (2 x 5min), 100% Ethanol (2 x 5mins), 70% Ethanol (1 x 5min) and DEPC-treated water (1 x 5min) to de-wax. Sections were then further washed in DEPC-treated water (2 x 2min) and stained with 1% Nuclear Fast Red (2min), so that nuclei were visible under a light microscope, followed by two further 2 min washes of DEPC-treated water. All solutions, except 1% Nuclear Fast Red, were changed every two cases or more often if necessary. After staining, slides were then gently tapped on a fresh piece of paper tissue in order to remove excess water, taking care not to over-dry the sections.

Micro-dissection

Prior to micro-dissection, all surfaces, including microscope, surrounding bench, forceps and containers were thoroughly cleaned as described previously. For each sample a barcode labelled cryovial pre-filled with 240µl of PKD buffer was prepared. Individual NFR-stained sections were microscopically examined and, using the marked H&E as a guide, the areas of invasive tissue micro-dissected. This was achieved by using the tip of a 19G sterile needle to carefully lift the tissue into the PKD buffer containing cryovial. Once all sections from the case had been micro-dissected, the cryovial was vortexed (15 sec) to ensure adequate mixing. The cryovial was then immediately snap-frozen on dry ice and transferred to -80^{0}C storage until shipping.

Preparation of Materials

DEPC-Treated Water: 500ml Duran bottles were filled with deionised water and 500µl of Diethyl Pyrocarbonate (DEPC) solution (SIGMA D5758-100ml) was added to each bottle in a fume hood and mixed well by inverting several times. Bottles were then left in the fume hood overnight to allow excess fumes to evaporate, before being put through two autoclave sterilization cycles. Once cooled the DEPC-treated water was ready for use.

Sterilised Glass Slides: Uncharged slides were put through two 5 minutes washes, first in 100% Ethanol and then in DEPC-treated water, before being placed into a slide box pre-cleaned with RNase Zap (Ambion cat. no. AM9780). Slides were then exposed to UV light for a minimum of 20 minutes after which the box was sealed with autoclave tape ready for use.

1% Nuclear Fast Red Solution: A 1% Nuclear Fast Red solution was made up by adding 50g of Aluminium Sulphate powder (Fisher cat. no. A/2600/53) to a beaker containing 1L of DEPC-treated water and allowing it to dissolve on a stirring hot plate heated to 100^{0}C. Once fully dissolved, 1g of NFR powder (Sigma cat. no. 60700) was added to the solution and also allowed to dissolve. The solution was left to cool down before being filtered using two 500ml Vacuum Filtration (TPP 99500) and then stored in at 4^{0}C ready for use.

Barcoded Cryovials: 2ml cryovials were labelled using barcodes provided by Gen-Probe Life Sciences Ltd, the company performing the total RNA extraction, and pre-filled with 240µl of PKD buffer (Qiagen RNeasy FFPE kit cat. no. 73504).

Tissue selection

All FFPE tumour blocks from the study cohort were selected from the archive and an HE stained section prepared for pathological review. Only sections demonstrating invasive carcinoma of more than $20mm^2$ (either a single area or multiple areas) were retained. Areas of invasive carcinoma were outlined on the coverslip during microscopical examination. Care was taken to avoid non-invasive malignant cells, lymphocytes and normal cells. The number of 8μm sections to be cut from each case for RNA extraction was based on the total area and cellularity of invasive cells marked on the section. Overloading the RNA extraction kit column with too much starting material has been shown to have a detrimental effect on RNA yield (1) so an algorithm based on a previous study (2) was used to validate the extraction kit.

RNA extraction and quality assessment

Samples were sent in batches to Gen-Probe Life Sciences Ltd to carry out RNA extraction using the Qiagen RNeasy FFPE kit (cat. no. 73504). RNA samples were returned with accompanying data on quality and quantity. RNA was quantified using a NanoDrop spectrophotometer reporting A260/A280 and A260/A230 ratios, concentration and yield, and RiboGreen (Invitrogen) reporting concentration and yield. RNA was assessed using Ct values (qRT-PCR of RPL13a) and RIN values (Agilent 2100 Bioanalyser). A normalised sample (20uL of 50ng/uL) was prepared from each RNA sample in preparation for subsequent WG-DASL assay. RNA samples were taken forward for WG-DASL expression profiling based on Ct value. Samples with Ct < 29 were prioritised for WG-DASL (2-4) and a number of samples with Ct > 29 were included from each extraction batch. RNA sample selection was performed for each extraction batch based on Ct value and without reference to any other factors.

DASL labelling and microarray hybridisation

Total RNA was converted to cDNA using Illumina Whole-Genome DASL and hybridised onto Illumina HT-12 v4 BeadChips according to manufacturer’s instructions. WG-DASL hybridisation plates 1-7 were assayed using DASL reagent MCS3, and WG-DASL hybridisation plates 8-15 using MCS4+RTE. Microarrays were scanned using the Illumina iScan system. Samples were mixed across BeadChips and array positions (A-L) to avoid confounding by date of diagnosis, ER status, tumour grade, and metastatic group (5, 6).

Microarray data processing

Raw intensity data (idat files) were imported into Illumina GenomeStudio (version 2011.1, GE v1.9.0) with no background correction (7). Probes with missing data were excluded within each hybridisation plate (96 arrays) to avoid introducing imputation effects. Bead summary data were exported from GenomeStudio with no further processing and imported into R/Bioconductor using the 'beadarray' package (8). The subset of probe IDs common to each hybridisation plate were combined to form two data sets corresponding to the two different DASL reagents: arrays from hybridisation plates 1-7 (29,632 probe IDs) and plates 8-15 (27,165 probe IDs). Probe annotations were assigned using the R/Bioconductor package 'illuminaHumanWGDASLv4.db' (v1.18.0) (9). Ensembl Gene ID coverage was 19,167 unique Ensembl Gene IDs for hybridisation plates 1-7 and 18,148 for plates 8-15. The majority of arrays from hybridisation plates 6-7 were removed from further analysis (due to issues with the platform reagent) and a subset of samples assayed on hybridisation plates 4-7 were repeated on plates 8-15. Outlier arrays were excluded from further analysis following the inspection of raw intensity distributions. Arrays in *GWDa* (plates 1-7) were excluded if mean(log2 intensity) < 8.5 or standard deviation < 1.5. Arrays in *GWDb* (plates 8-15) were excluded if median(log2 intensity) < 8. The remaining arrays were quantile normalised within *GWDa* and *GWDb*. Samples present in both *GWDa* and *GWDb* were removed from *GWDa* where it was used as a validation set for gene expression scores. Putative probe-level batch effects (6) which may be related to BeadChip, hybridisation or extraction batches were inspected. Principal component analysis indicated significant associations between dominant components and ER status, tumour grade and metastatic group. In addition, there were significant components associated with RNA extraction batch and chip position. After applying a gene-specific linear adjustment (ComBat (10, 11)), the associations with ER and grade were weakened while technical factors were still associated with several principle components of the adjusted data. Therefore, in this study we worked with quantile normalised data with no further manipulation for technical correlates at the preprocessing stage. Probes with the largest technical batch effects were identified for reference.

Assessment of quantity and quality of RNA extracted from long-term stored FFPE material

Extracted RNA was inspected before proceeding to WG-DASL. RNA extracted from FFPE material show varying degrees of degradation (2-4). We investigated whether factors related to sample storage, the age of the tissue block and the type of fixative used, affected the quantity and quality of extracted RNA. The RNA samples in this study were from patients diagnosed between 1975 and

2005, and tissue samples were dissected fresh then fixed using one of three formalin-based fixatives (formol saline, formol calcium, phenol formol saline) according to the protocol in place at the date of primary diagnosis and subsequent tissue storage (Supplementary Figure 6A). RNA samples from lymph node metastases spanned the same period of diagnosis times (Supplementary Figure 6B). Increased RNA yield and purity as measured by A260/A280 ratio (optimal value ≈ 2) from primary tumour samples was associated with shorter storage time (Supplementary Figure 6). RNA yield and purity from lymph node samples was not as sensitive to storage time as the primary tumour samples. Lymph node metastases produced similar RNA yields and purity across all time periods of diagnosis and higher yields than primary tumour samples (lymph node, median: 13.4µg; primary tumour, median: 9.1µg; p=8x10^{-7}, Mann-Whitney *U*) (Supplementary Figure 6D,F).

RNA quality measured by Ct value (RPL13a Q-PCR) indicated overall poorer RNA quality (higher Ct values) for primary tumour samples with longer storage times (Supplementary Figure 6G). A similar loss of RNA quality with storage time was observed for lymph node metastases; however, the oldest lymph node samples (1975-1990) were overall of higher quality than primary tumour samples of similar age (Supplementary Figure 6H).

RNA yield and quality (Ct value) were compared with tumour pathological factors within three decade-long periods of diagnosis, due to the expected effect of storage time on RNA quantity and quality (Supplementary Figure 6I,J). Tumour invasive grade was associated with RNA yield within all three decades of diagnosis (1975-1984: p=2x10^{-6}; 1985-1994: p=7x10^{-12}; 1995-2005: p=8x10^{-9}; Kruskal-Wallis) (Supplementary Figure 6I). Estrogen receptor (ER)-negative tumours produced slightly higher median RNA yields but this was significant only within the samples of shortest storage time (1975-1984: p=0.1; 1985-1994: p=0.04; 1995-2005: p=0.005; Mann-Whitney *U*) (Supplementary Figure 6I). Similarly, triple negative breast tumours (TNBC; IHC ER-, PgR- and HER2-negative) produced higher median yields than hormone receptor-positive tumours (at least one of IHC ER, PgR or HER2 known and positive) with a significant difference within the most recent decade (1975-1984: p=0.6; 1985-1994: p=0.3; 1995-2005: p=0.002; Mann-Whitney *U*) (Supplementary Figure 6I). There was no evidence of corresponding higher RNA quality (lower Ct values) in TNBCs (Supplementary Figure 6J).

Design and preparation of WG-DASL gene expression data sets

RNA samples were selected for WG-DASL assays based on Ct value, as previous studies report that Ct value may be a useful indicator for proceeding to WG-DASL (3, 4). RNA samples with Ct < 29 were prioritised for WG-DASL and a number of samples with Ct > 29 were included to test their feasibility (Supplementary Figure 6).

In total, 874/1,370 primary tumour RNA samples and 79/100 lymph node metastasis RNA samples were taken forward to WG-DASL assay. Sample characteristics including metastatic group, ER status and grade were distributed across BeadChips and randomised with respect to array position (A-L) (6). To assess the technical variability of the WG-DASL assay, technical duplicates of ten samples were included on each hybridisation plate (96-well plate), and across different hybridisation plates.

Hybridisation plates 1-7 and 8-15 were analysed as separate data sets due to a change in WG-DASL kit reagent (see Methods; Fig 1). Overall, WG-DASL success rate and data quality was improved for hybridisation plates 8-15 compared to plates 1-7. Samples on plates 8-15 were therefore used to construct the primary training set (Guy's WG-DASL, "*GWDb"*) while samples on plates 1-7 were used as the validation set ("*GWDa"*).

The primary tumour and lymph node metastasis samples taken forward to hybridisation were found to represent the full range of storage times. Ct distributions varied with storage time (Supplementary Figure 6C-D) and higher Ct values corresponded to a modest overall decrease in array intensities (Supplementary Figure 6G). Inspection of Ct value and density of RNA fragment lengths suggests that array failure/lower quality has a modest association with increased Ct value and reduced density of fragments around 200nt (as determined by Agilent Bioanalyser 2100 Expert software); however, neither of these RNA diagnostics were discriminatory for array failure/lower quality in this data set (Supplementary Figure 6H).

Assessment of reproducibility across technical replicates

The reproducibility of sample profiles was established by inspecting (i) the correlation of profiles from technical duplicates, (ii) the consistency of molecular subtype assignments (based on the PAM50 classifier (12)), and (iii) the expression profiles derived from cell line samples. The reproducibility of technical duplicates (two WG-DASL assays for a single extracted RNA sample) was investigated by calculating pairwise Pearson correlation coefficients for all samples. The mean (range) of correlation for technical duplicates was 0.98 (0.88-0.9959), compared with pairwise

correlations between all other samples, median 0.91 (0.44-0.98), demonstrating high specificity of similarity between technical duplicates (Supplementary Figure 6E; $p<10^{-16}$, Mann-Whitney *U*). Within the range of technical duplicate correlations, higher correlation was associated with lower Ct values and higher RNA yield, but did not show an association with the density of fragments with length 150-200nt (Supplementary Figure 6E). Gene expression profiles of duplicated RNA assays from the same patients were assigned to molecular breast cancer subtypes using the PAM50 centroid classification (12), with 72 out of the 82 cases in *GWDb* (88%) and 21/21 (100%) in *GWDa* classified to the same molecular subtypes.

To further explore the quality of the *GWDb* data set, cell line expression profiles were assessed for whether consistent profiles could be recovered from the same cell line samples distributed across different hybridisation plates. FFPE samples from six breast cancer cell lines were assayed in technical duplicate, and cell line samples within a given duplicated pair were assayed on different hybridisation plates and preprocessed within data set *GWDb*. An unsupervised clustering of highly variable probes from the resulting expression profiles showed that all technical duplicates clustered together (with the exception of HCC11954 which array failed the initial quality assessment) and separated the basal-like from the luminal-like cell lines (13, 14).

Overlaps with other gene expression data sets

A number of patients in this study overlap with other studies, as follows:

**Study(s)**	**Reference(s)**	**Total patient overlap (extracted RNA)**	**Total patient overlap (*GWDb*)**
METABRIC	Curtis *et al.* 2012 [**ref (15)**]	63	40
Breakthrough studies	Braso-Maristani *et al.* 2016 [**ref (16)**]	34	15

ER status for stratified analyses

We aimed to avoid potential contamination of WG-DASL derived ER-positive or ER-negative data sets where individual samples appear to have a discrepancy between IHC ER status and ESR1 expression. To avoid a potential confounding factor related to discrepancy between IHC ER status

(clinico-pathological information) and ESR1 expression levels (WG-DASL), samples were considered “ER-positive” if both IHC status and ESR1 gene expression level (ESR1 > 10.3) indicate ER-positivity. Samples with a discordant IHC and ESR1 gene expression level were excluded from ER stratification. The threshold for ESR1 was identified on inspection of bimodal density plots of ESR1 expression level across samples, and then tested to verify that the number of discordant samples was close to the minimum possible when compared with other thresholds. This definition also reduces the possibility that IHC ER-positive samples show an inflation in the number of samples with *“visceral only”* metastases, and the ER-negative group an inflation in *“bone only”* metastases, if samples are identified as ER-positive or ER-negative on the basis of IHC status alone.

Gene module scoring

Scores were assigned using DART (17) and further compared with weighted sum (weights (+1,-1) according to the direction of expression in the gene signature) except for SRC response which was modelled as a binary variable. Previously reported gene expression signatures were mapped to WG-DASL probes using Ensembl Gene ID, Entrez Gene ID or gene symbol, according to their original source (Table S2). Where multiple microarray probes mapped to a single Entrez Gene ID, the probe with the most variable gene expression across the datasets was used (based on standard deviation in the relevant data set). SRC response was modelled as a binary variable: primary tumour samples with an active SRC response (SRC+) were defined as samples within the upper tertile of SRC gene expression in GWDb (patient de-duplicated).

Supplementary References

1. Qiagen. http://www.qiagen.com/products/catalog/sample-technologies/rna-sample-technologies/total-rna/rneasy-ffpe-kit#resources. Qiagen RNeasy FFPE Handbook.
2. Abramovitz M, Ordanic-Kodani M, Wang Y, Li Z, Catzavelos C, Bouzyk M, et al. Optimization of RNA extraction from FFPE tissues for expression profiling in the DASL assay. BioTechniques. 2008;44:417-23.
3. April C, Klotzle B, Royce T, Wickham-Garcia E, Boyaniwsky T, Izzo J, et al. Whole-genome gene expression profiling of formalin-fixed, paraffin-embedded tissue samples. PLoS ONE. 2009;4:e8162.
4. Waddell N, Cocciardi S, Johnson J, Healey S, Marsh A, Riley J, et al. Gene expression profiling of formalin-fixed, paraffin-embedded familial breast tumours using the whole genome-DASL assay. J Pathol. 2010;221:452-61.
5. Verdugo RA, Deschepper CF, Munoz G, Pomp D, Churchill GA. Importance of randomization in microarray experimental designs with Illumina platforms. Nucleic Acids Res. 2009;37:5610-8.

6. Kitchen RR, Sabine VS, Sims AH, Macaskill EJ, Renshaw L, Thomas JS, et al. Correcting for intra-experiment variation in Illumina BeadChip data is necessary to generate robust gene-expression profiles. BMC Genomics. 2010;11:134.
7. Dunning MJ, Barbosa-Morais NL, Lynch AG, Tavare S, Ritchie ME. Statistical issues in the analysis of Illumina data. BMC Bioinformatics. 2008;9:85.
8. Dunning MJ, Smith ML, Ritchie ME, Tavare S. beadarray: R classes and methods for Illumina bead-based data. Bioinformatics. 2007;23:2183-4.
9. Barbosa-Morais NL, Dunning MJ, Samarajiwa SA, Darot JF, Ritchie ME, Lynch AG, et al. A re-annotation pipeline for Illumina BeadArrays: improving the interpretation of gene expression data. Nucleic Acids Res. 2010;38:e17.
10. Johnson WE, Li C, Rabinovic A. Adjusting batch effects in microarray expression data using empirical Bayes methods. Biostatistics. 2007;8:118-27.
11. Leek JT, Johnson WE, Parker HS, Jaffe AE, Storey JD. The sva package for removing batch effects and other unwanted variation in high-throughput experiments.; 2012.
12. Parker JS, Mullins M, Cheang MC, Leung S, Voduc D, Vickery T, et al. Supervised risk predictor of breast cancer based on intrinsic subtypes. J Clin Oncol. 2009;27:1160-7.
13. Neve RM, Chin K, Fridlyand J, Yeh J, Baehner FL, Fevr T, et al. A collection of breast cancer cell lines for the study of functionally distinct cancer subtypes. Cancer Cell. 2006;10:515-27.
14. Grigoriadis A, Mackay A, Noel E, Wu PJ, Natrajan R, Frankum J, et al. Molecular characterisation of cell line models for triple-negative breast cancers. BMC Genomics. 2012;13:619.
15. Curtis C, Shah SP, Chin S-F, Turashvili G, Rueda OM, Dunning MJ, et al. The genomic and transcriptomic architecture of 2,000 breast tumours reveals novel subgroups. Nature. 2012:1-7.
16. Braso-Maristany F, Filosto S, Catchpole S, Marlow R, Quist J, Francesch-Domenech E, et al. PIM1 kinase regulates cell death, tumor growth and chemotherapy response in triple-negative breast cancer. Nat Med. 2016;22:1303-13.
17. Jiao Y, Lawler K, Patel GS, Purushotham A, Jones AF, Grigoriadis A, et al. DART: Denoising Algorithm based on Relevance network Topology improves molecular pathway activity inference. BMC Bioinformatics. 2011;12:403.

Sweave: conditional logistic regression and logistic regression models of computed gene module scores

```r
## Based on script do_CCseries_sigPanelOR_fromcCTable.R, git commit bed7f895

# Read the case-control expanded table (by RS;c/c) with appended columns of
# covariables (matched by Patient.ID) sigScores as cts variables: Perform
# (univar) conditional OR tests with RS_id's as strata (allow one intersept
# per RS_id)

library(Epi)  # for clogistic, conditional logistic regression


# Utility function x is a numeric vector, vector of sig scores Return a
# vector of the same length, where (5th,95th) quantiles are set to approx
# (-1,1)
shrink <- function(x, upper = 0.95, lower = 0.05) {
    x <- x - median(x, na.rm = TRUE)
    x <- 2 * x/(quantile(x, upper, na.rm = TRUE) - quantile(x, lower, na.rm = TRUE))
    return(x)
}



## Read the case-control expanded table (by RS;c/c) with appended columns of
## covars/annotation

mdat <- read.delim(file = "../output/master_mdat_byRS_hasExtractedCase_byResIDintentions__expandRScC_annot.txt",
    sep = "\t", header = TRUE)


## OR tests for each sig score, in GWDb GWDb_sigs: cts with RS_id strata,
## conditional logistic regression using Epi::clogistic

sigNames <- c(grep("^GWDb__sigScore2DART_", colnames(mdat), value = TRUE), "GWDb__SRCpos")

## Example: sigName <- sigNames[1] clogistic(
## factor(mdat$Case_Control,levels=c('Control','Case')) ~ mdat[,sigName],
## strata=as.factor(mdat$Random_Selection) ) clogistic(formula, strata, data,
## subset, na.action, init, model = TRUE, x = FALSE, y = TRUE )



do_CondLogReg_bySig <- function(dat, sigName, caseType = "", ERtype = "") {
    # caseType: V,BV,B,[anything else doesn't subset]; ERtype:
    # ERpos,ERneg,[anything else doesn't subset]

    # Filter RS's by caseTypes
    keepRS <- unique(dat$Random_Selection)  # Init, Vector of RS values to be included
    if (caseType %in% "B") {
        keepRS <- unique(dat$Random_Selection[dat$CaseType %in% "Bone Only"])
    }
    if (caseType %in% "V") {
        keepRS <- unique(dat$Random_Selection[dat$CaseType %in% "Visceral Only"])
    }
    if (caseType %in% "BV") {
        keepRS <- unique(dat$Random_Selection[dat$CaseType %in% "Bone+Visceral"])
    }
    dat <- dat[(dat$Random_Selection %in% keepRS), ]
    rm(keepRS)


    ## Filter RS's by ER status of case AND control
    if (ERtype %in% "ERpos") {
        # Keep only the entries which are of this ERtype
        dat <- dat[(dat$GWDb__ER_ESR1_IHC %in% 1), ]  # Note: excludes any samples in plates1to7 only (NA)

        # Keep only paired RS's. The remaining entries are RS-pairs of this ERtype.
        dat <- dat[dat$Random_Selection %in% dat$Random_Selection[duplicated(dat$Random_Selection)],
            ]
    }


    if (ERtype %in% "ERneg") {
        # Keep only the entries which are of this ERtype
        dat <- dat[(dat$GWDb__ER_ESR1_IHC %in% 0), ]  # Note: excludes any samples in plates1to7 only (NA)

        # Keep only paired RS's. The remaining entries are RS-pairs of this ERtype.
        dat <- dat[dat$Random_Selection %in% dat$Random_Selection[duplicated(dat$Random_Selection)],
            ]
    }



    # Store which RS's are remaining in dat (used for the tests)
    RSused <- unique(dat$Random_Selection)

    # Cts, after rescaling to a standard scaling (within each sig) and to reduce
    # the effect of outliers (cf. Ben Haibe-Kains, JCO, 2012) This way, within a
```

```
    # dat.expand (case/control selection), cts sig OR's are comparable (OR's for
    # a unit increase in a scaled module score)
    dat[, sigName] <- shrink(dat[, sigName])


    mylogit.cts.cond <- clogistic(factor(dat$Case_Control, levels = c("Control",
        "Case")) ~ dat[, sigName], strata = as.factor(dat$Random_Selection))

    dat.cond <- dat  # Keep

    dat.wilcox <- dat[dat$Case_Control %in% "Case", c("Random_Selection", sigName)]
    dat.controls <- dat[dat$Case_Control %in% "Control", ]
    dat.wilcox <- cbind(dat.wilcox, dat.controls[match(dat.wilcox$Random_Selection,
        dat.controls$Random_Selection), sigName])
    colnames(dat.wilcox)[2:3] <- paste(c("Case", "Control"), sigName, sep = ".")
    rm(dat.controls)

    res.wilcox <- wilcox.test(dat.wilcox[, paste("Case", sigName, sep = ".")],
        dat.wilcox[, paste("Control", sigName, sep = ".")], paired = FALSE)
    res.wilcox.paired <- wilcox.test(dat.wilcox[, paste("Case", sigName, sep = ".")],
        dat.wilcox[, paste("Control", sigName, sep = ".")], paired = TRUE)

    cts.summary.Cases <- summary(dat.wilcox[, paste("Case", sigName, sep = ".")])
    cts.summary.Controls <- summary(dat.wilcox[, paste("Control", sigName, sep = ".")])


    # For unconditional (unpaired), remove duplicate patients from the controls
    dat <- dat[!duplicated(paste(dat$Case_Control, dat$Patient.ID)), ]
    mylogit.cts <- glm(factor(dat$Case_Control, levels = c("Control", "Case")) ~
        dat[, sigName], family = "binomial")

    dat.uncond <- dat[, c("Random_Selection", "Case_Control", "GWDb__PAM50.Nearest.centroid",
        "Patient.ID")]  # Keep

    return(list(mylogit.cts = mylogit.cts, mylogit.cts.cond = mylogit.cts.cond,
        RS.cond = RSused, res.wilcox = res.wilcox, res.wilcox.paired = res.wilcox.paired,
        cts.summary.Cases = cts.summary.Cases, cts.summary.Controls = cts.summary.Controls,
        dat.cond = dat.cond, dat.uncond = dat.uncond, dat.wilcox = dat.wilcox))

}




################# For each caseType
for (caseType in c("anyCaseType", "V", "BV", "B")) {

    ## Capture output to file
    out.file <- paste("output/sigPanel_condLogRegORs_", caseType, "_byInt.txt",
        sep = "")
    sink(out.file)

    # Init results stores
    resListc <- list()  # clogistic output object
    resListc.summary <- list()  # summary(clogistic output)
    resListc.OR <- list()  # OR, 95% CI

    # Init results stores
    resListcGGIadj <- list()  # clogistic output object
    resListcGGIadj.summary <- list()  # summary(clogistic output)
    resListcGGIadj.OR <- list()  # OR, 95% CI



    # ERtype <- 'ERany'
    for (ERtype in c("ERany", "ERpos", "ERneg")) {
        for (sigName in sigNames) {
            print("--------------------------------------------------------------")
            print(ERtype)
            print(caseType)
            print(sigName)

            if (!((ERtype %in% "ERneg") & (caseType %in% c("V", "B")))) {
                res <- do_CondLogReg_bySig(dat = mdat, sigName = sigName, caseType = caseType,
                  ERtype = ERtype)

                print(res$mylogit.cts.cond)

                # Assign results
                resListc[[ERtype]][[sigName]][["mylogit.cts.cond"]] <- (res$mylogit.cts.cond)
                resListc.summary[[ERtype]][[sigName]][["mylogit.cts.cond"]] <- summary(res$mylogit.cts.cond)
                resListc.OR[[ERtype]][[sigName]][["mylogit.cts.cond"]] <- exp(cbind(OR = coef(res$mylogit.cts.cond),
                  confint(res$mylogit.cts.cond)))

                resListc[[ERtype]][[sigName]][["mylogit.cts"]] <- (res$mylogit.cts)
                resListc.summary[[ERtype]][[sigName]][["mylogit.cts"]] <- summary(res$mylogit.cts)
                resListc.OR[[ERtype]][[sigName]][["mylogit.cts"]] <- exp(cbind(OR = coef(res$mylogit.cts),
                  confint(res$mylogit.cts)))
```

```
                resListc[[ERtype]][[sigName]][["res.wilcox"]] <- (res$res.wilcox)
                resListc[[ERtype]][[sigName]][["res.wilcox.paired"]] <- (res$res.wilcox.paired)
                resListc[[ERtype]][[sigName]][["cts.summary.Cases"]] <- (res$cts.summary.Cases)
                resListc[[ERtype]][[sigName]][["cts.summary.Controls"]] <- (res$cts.summary.Controls)

                resListc[[ERtype]][[sigName]][["RS.cond"]] <- res$RS.cond

                resListc[[ERtype]][[sigName]][["mylogit.cts.cond_dat"]] <- (res$dat.cond)
                resListc[[ERtype]][[sigName]][["mylogit.cts_dat"]] <- (res$dat.uncond)
                resListc[[ERtype]][[sigName]][["wilcox_dat"]] <- (res$dat.wilcox)


                rm(res)
            }

        }
    }



    sink()


    ########################## Export

    save(list = c("resListc", "resListc.OR", "resListc.summary"), file = paste("output/res_sigPanel_condLogRegORs_",
        caseType, "_byInt.rda", sep = ""))

}

sessionInfo()
```
