# Supplementary material for: Gene expression modules in primary breast cancers as risk factors for organotropic patterns of first metastatic spread: a case control study
Source: Breast Cancer Res. 2017 Oct 13;19:113. doi: 10.1186/s13058-017-0881-y (PMC5640935; doi:10.1186/s13058-017-0881-y)
Supplement: Supplementary file 2 — Figures S1-S8.. (PDF 2279 kb) [file 13058_2017_881_MOESM2_ESM.pdf]

**Supplementary Figure S1.** Overview flowchart of patient selection, RNA extraction, sample selection and generation of WG-DASL gene expression data sets.

**A.** Case-control sampling was performed for three metastatic groups: *'bone only'* (first recurrence to bone only without any other metastatic site within 6 months, *'bone & visceral'* (within a 6 month period) and *'visceral only'* (first recurrence to visceral only without any other metastatic site within 6 months). Case-control pairs were randomly selected for inclusion and each selected tissue block was assessed for RNA extraction. RNA was extracted from 1,370 primary tumour samples, 100 patient-matched lymph node metastases, and six cell line samples. Following RNA quality control, WG-DASL quality assessment and data processing, two gene expression data sets were produced (GWDb, GWDa). (Part **B** continues on the next page).

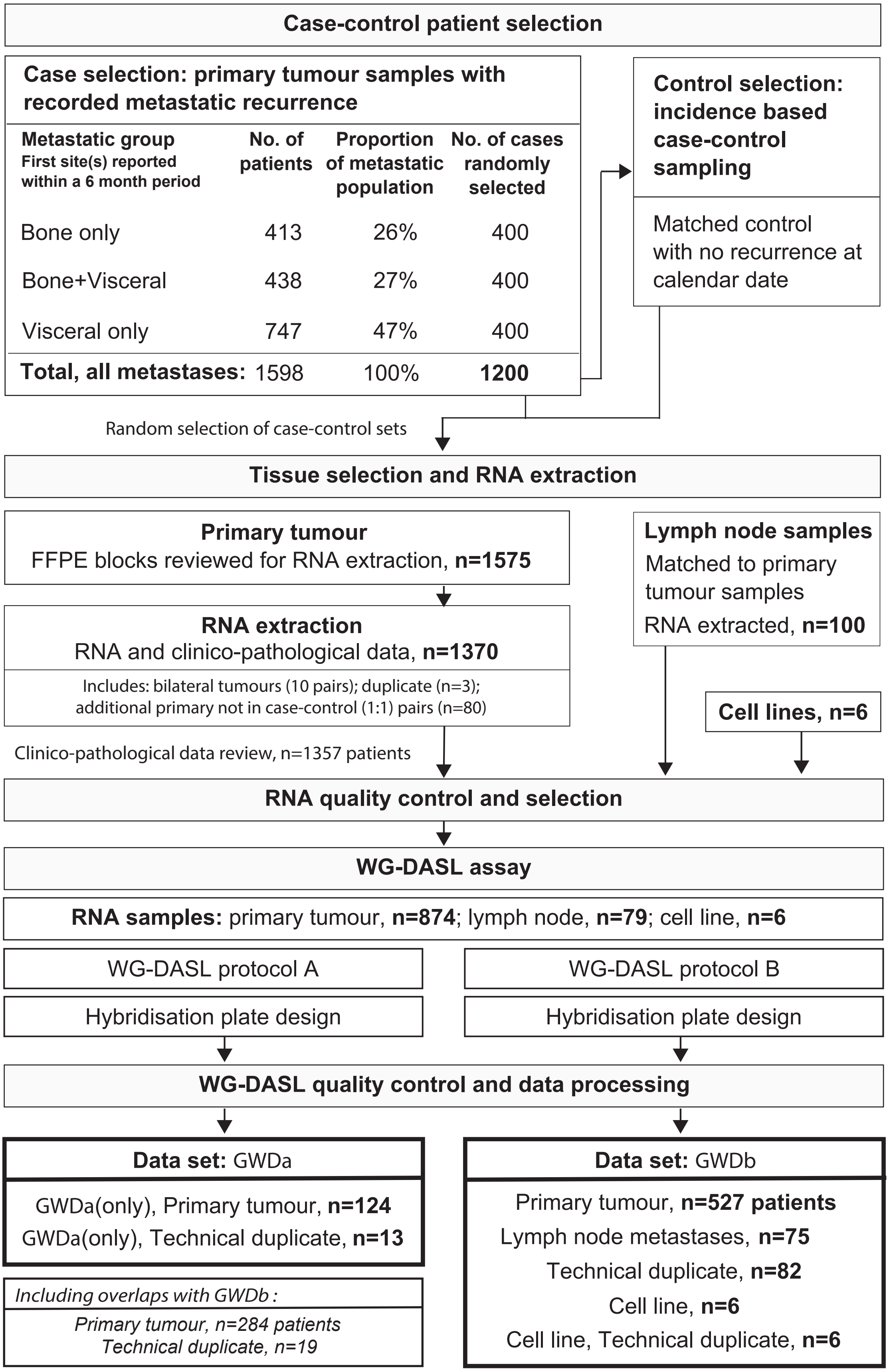

**Supplementary Figure S1 (continued).**
**B.** Case-control sampling and the number of cases available among extracted RNA and within the gene expression data sets.

**Study metastatic population**

| Metastatic group<br>First site(s) reported within a 6 month period | No. of patients | Proportion of metastatic population |
|---|---|---|
| Bone only | 413 | 26% |
| Bone+Visceral | 438 | 27% |
| Visceral only | 747 | 47% |
| **Total, all metastases:** | 1598 | 100% |

Incidence-based case-control sampling
1598 sample sets (1 case + 3 possible controls)

**Random selection of cases of each type**

| | No. of patients | Proportion of selected cases |
|---|---|---|
| Bone only | 400 | 33% |
| Bone+Visceral | 400 | 33% |
| Visceral only | 400 | 33% |
| **Total, cases:** | 1200 | 100% |

1200 sample sets

Random selection of cases (any type) for tissue inspection and RNA extraction

**RNA extracted, cases (with extracted control)**

| | No. of patients | Proportion of selected cases |
|---|---|---|
| Bone only | 238 | 32% |
| Bone+Visceral | 258 | 35% |
| Visceral only | 246 | 33% |
| **Total, cases:** | 742 | 100% |

742 case-control pairs have case and control extracted RNA.
(1277 unique patients in assigned case-control pairs.)

Additional samples (see Fig S1A)

RNA quality control
WG-DASL quality control
Gene expression data processing
(see Fig S1A)

| | **Within GWDb, cases, unpaired** (i.e. ignoring control) | | **Within GWDa(only), cases, unpaired** | |
|---|---|---|---|---|
| | No. of patients | Proportion of selected cases | No. of patients | Proportion of selected cases |
| Bone only | 98 | 32% | 19 | 31% |
| Bone+Visceral | 106 | 34% | 23 | 37% |
| Visceral only | 105 | 34% | 20 | 32% |
| **Total, cases:** | 309 | 100% | 62 | 100% |

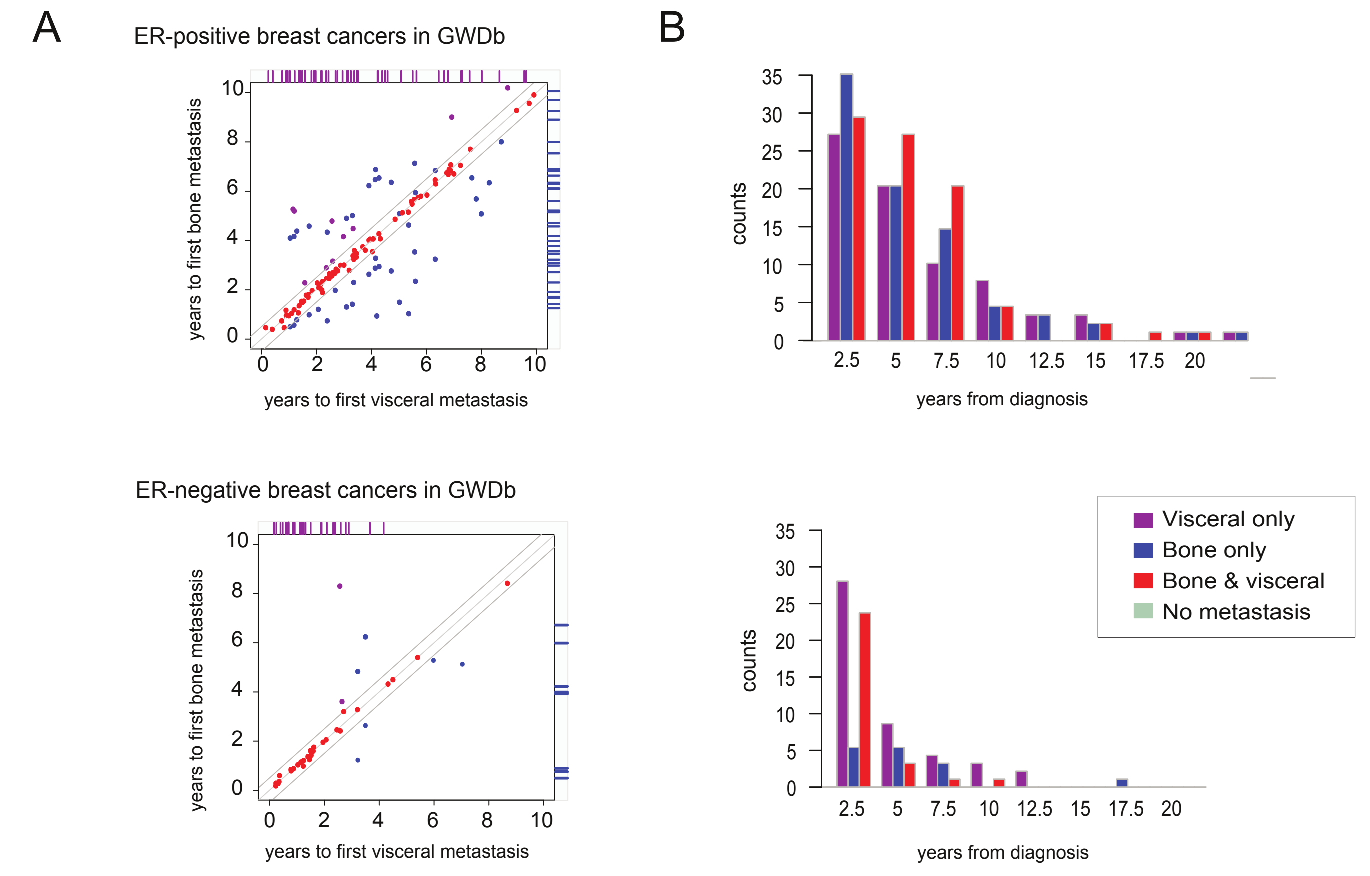


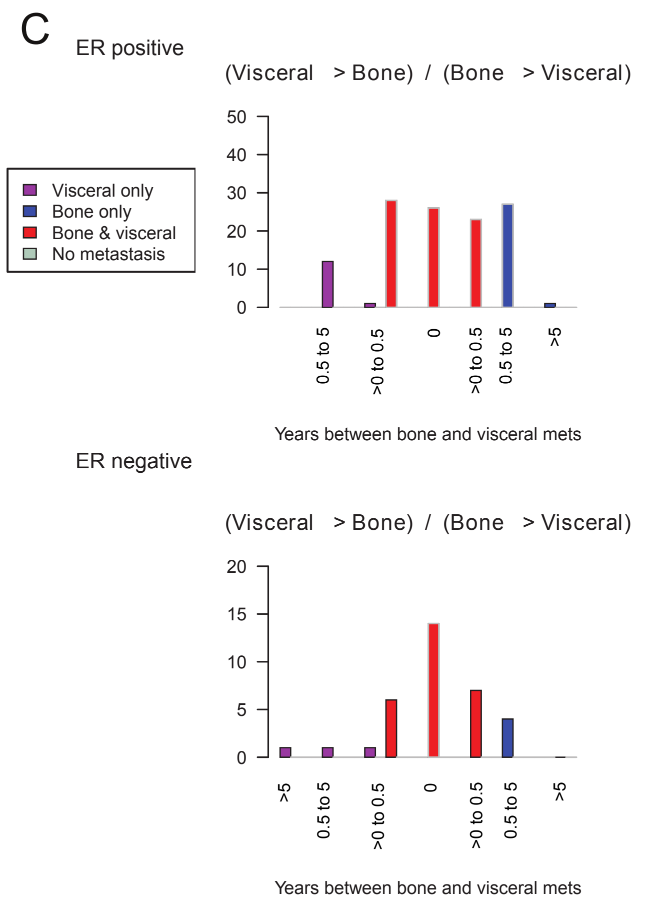


**Supplementary Figure S2.** Descriptive plots of data set *GWDb*: patterns of single metasynchronous multiple metastatic spread and long-term follow-up.

**A.** Time to bone or visceral metastases within 10 years of diagnosis. For those patients with both bone and visceral metastases recorded, the time to first visceral metastasis (y-axis) is displayed versus time to first bone metastasis, for ER-positive (top) and ER-negative (bottom) primary tumours (data set *GWDb*), separately. Along the y-axis (blue rug) are shown time to metastasis for those patients with only bone metastasis recorded, and along the x-axis those with only a visceral metastasis (magenta rug). Patients with bone and visceral metastases recorded within a period of six months are shown in red and comprise the *'bone & visceral'* metastatic group.

**B.** Barplots display the number of patients with 'bone only', *'visceral only'* and *'bone & visceral'* patterns of first metasynchronous metastases by time from primary diagnosis.

**C.** Barplots indicate the time between first recorded bone and first recorded visceral metastases. Bar colours indicate the definition of metastatic groups. In particular, the *'bone & visceral'* group comprises patients with bone and visceral metastases recorded within a six month time period.

**Supplementary Figure S3. An extended panel of gene modules with univariate logistic regression modelling of case-control series in *GWDb*: (A) including samples with missing paired samples; (B) for each case series versus all tumours with no metastasis; (C,D) exploratory modelling of time-to-site specific survival irrespective of case-control series.**

**A.** (i) Univariate logistic regression allowing incomplete pairs due to data missing from *GWDb*, where ER-positive/-negative data sets comprise ER-matched case-control pairs (*"caseGWDb[_bothERpos/neg]"*). Forest plots display log odds ratio (95% CI).
(ii) Univariate logistic regression allowing incomplete pairs, where ER-positive/-negative data sets comprise all individual cases and controls with the respective ER-status (not ER-matched) (*"caseGWDb[_ERpos/neg]"*). Forest plots display log odds ratio (95% CI).
*V*: "visceral-only" metastatic group; *BV*: "bone & visceral"; *B*:"bone-only"; *Mets*: any case type.
The number of samples available for each model is reported in Suppl Table S1. Fig. 3 displays conditional logistic regression (paired) and logistic regression models for illustrative gene modules.

**B.** Forest plots of odds ratios (logistic regression model) for each case series compared with all tumours with no recorded metastases.

**C-D.** Exploratory survival modelling of site-specific metastasis-free survival using gene modules (unscaled scores), for all patients with primary tumours in *GWDb*. Forest plots of hazard ratio (95% C.I.) estimated by fitting a univariate Cox proportional hazards model for site-specific metastasis-free survival for all ER-positive (C) and ER-negative (D) patients with primary tumours in data set *GWDb*, irrespective of the case-control series. Box sizes are inversely proportional to the size of the confidence interval.

## Supplementary Figure S3

**A**

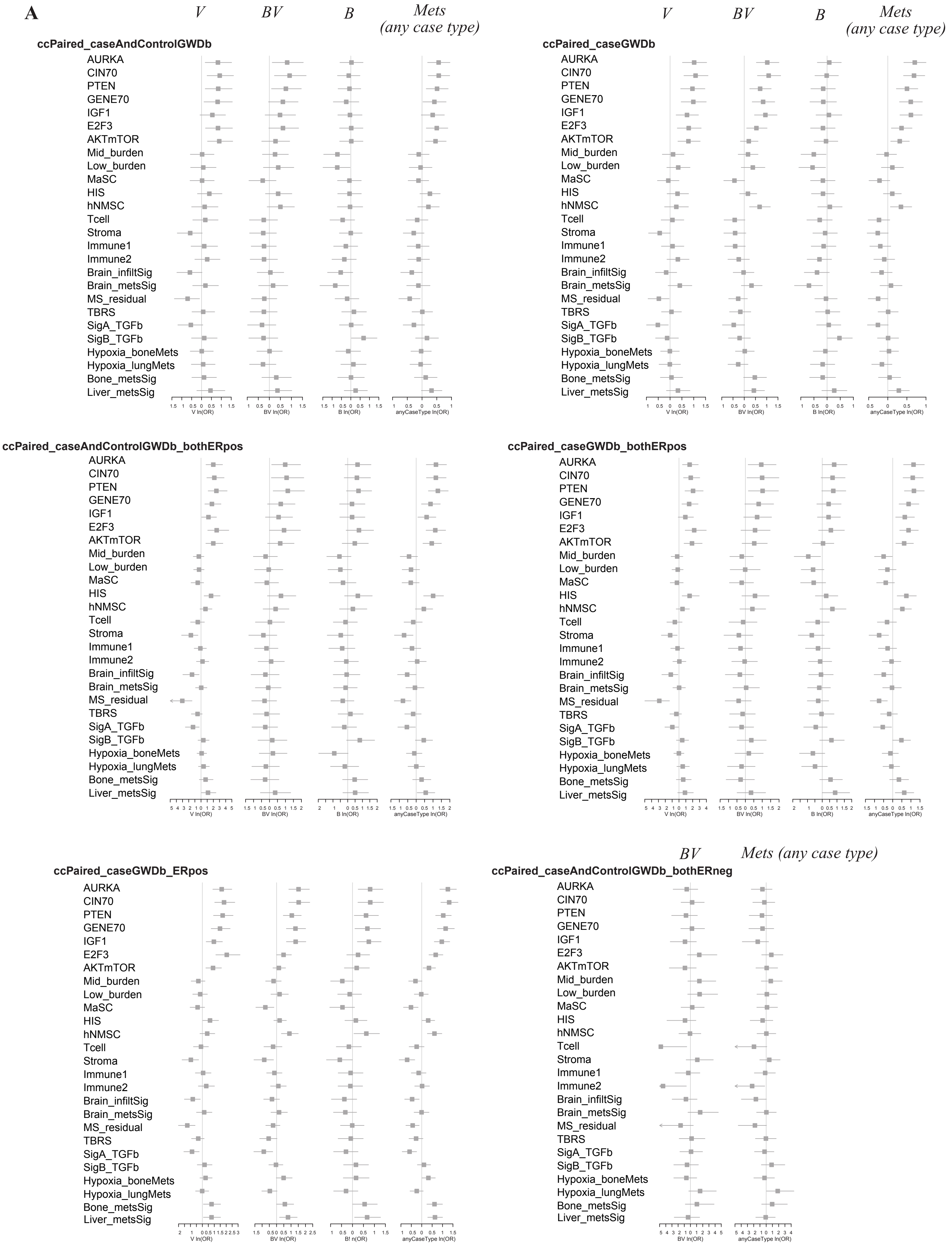


*Supplementary Figure S3 continues overleaf*

*Supplementary Figure S3 (continued)*

**A** *(continued)*

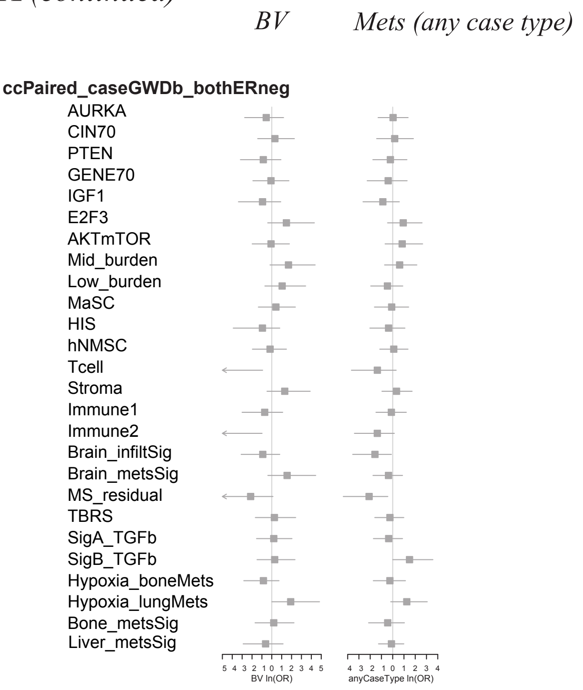


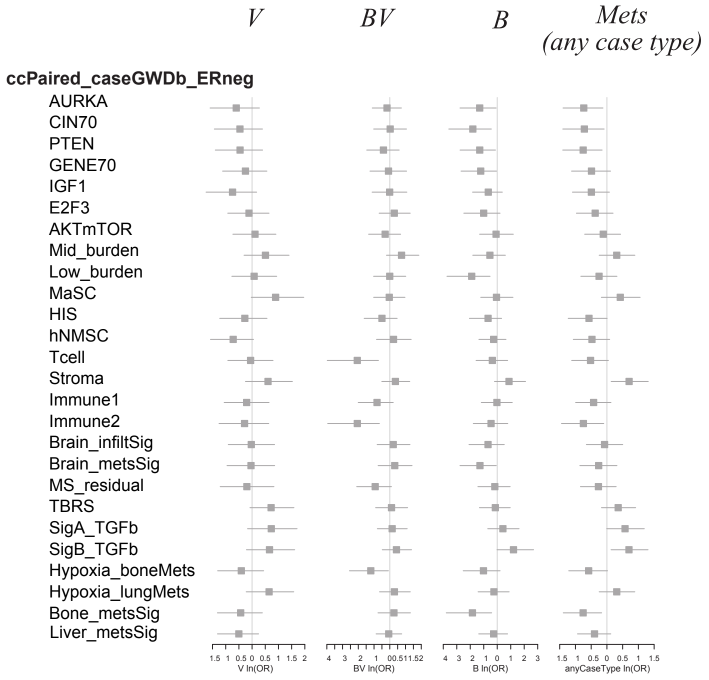


**B**

*Forest plots of odds ratios (logistic regression model) for each case series compared with all tumours with no recorded metastases.*

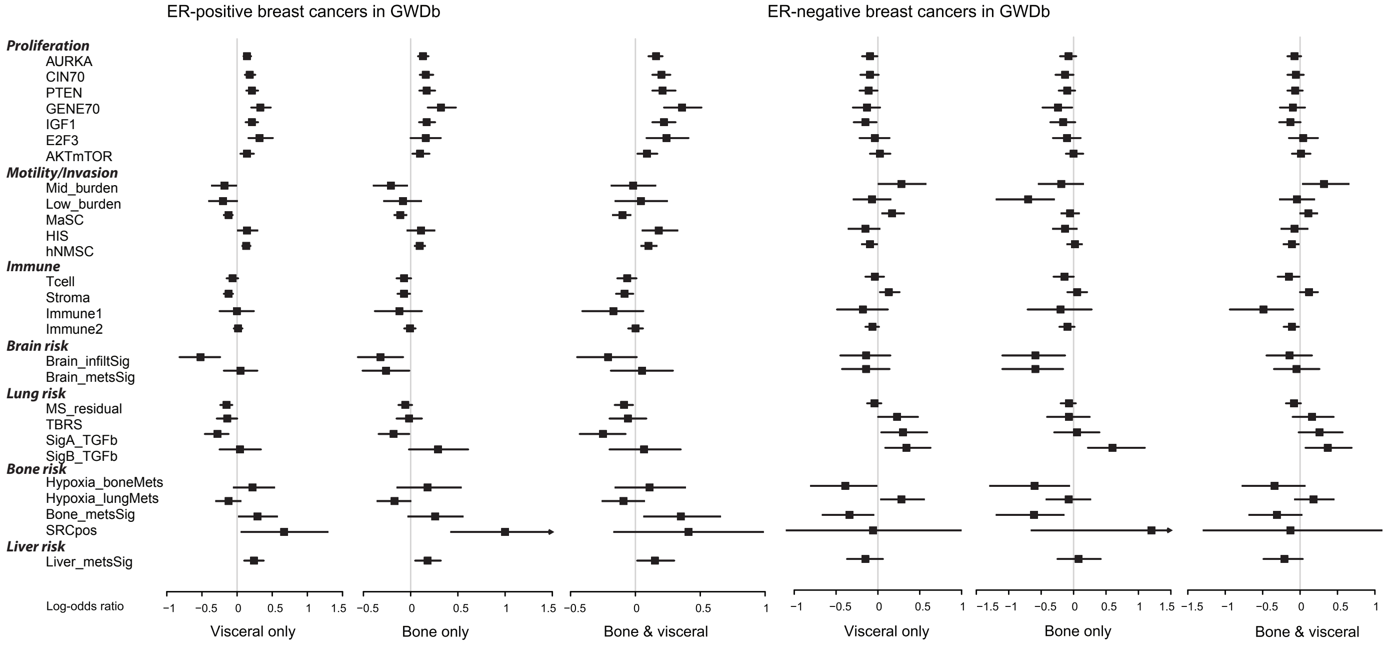


*Supplementary Figure S3 continues overleaf*

## Supplementary Figure S3 (continued)

### C

*ER-positive primaries, individual metastatic sites*
*Cox survival model, hazard ratio (95% CI)*

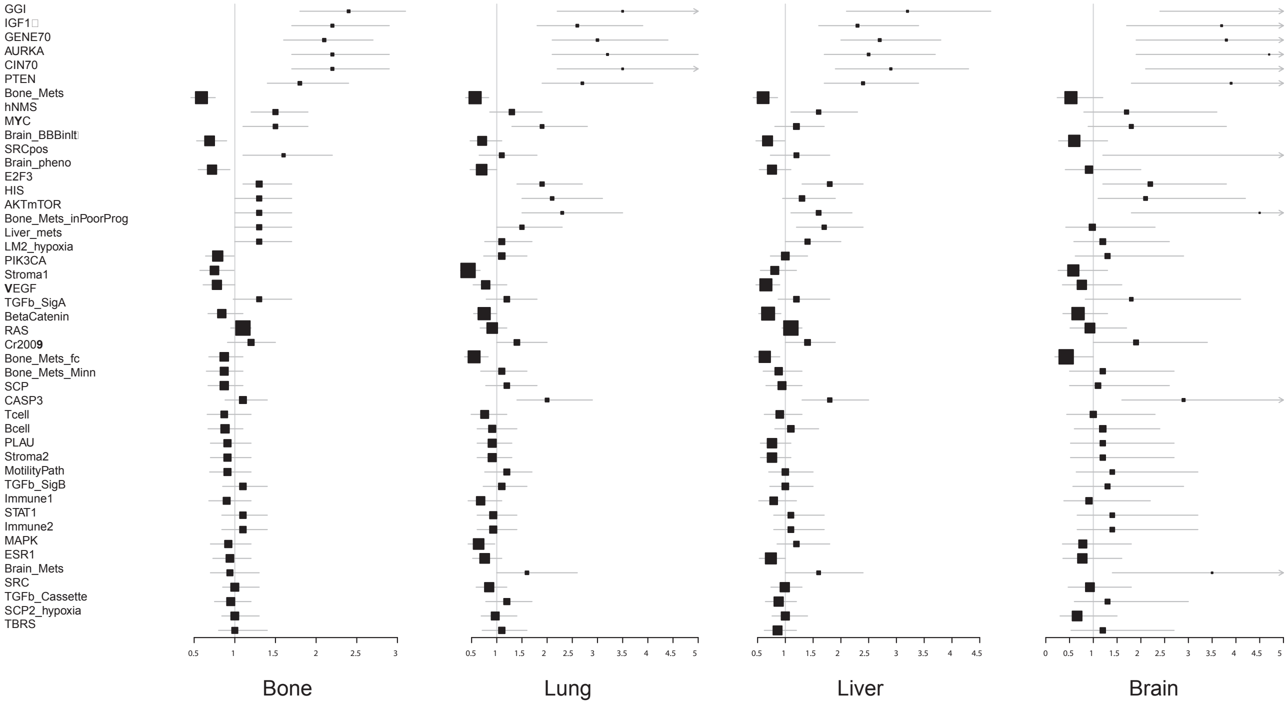


### D

*ER-negative primaries, individual metastatic sites*
*Cox survival model, hazard ratio (95% CI)*

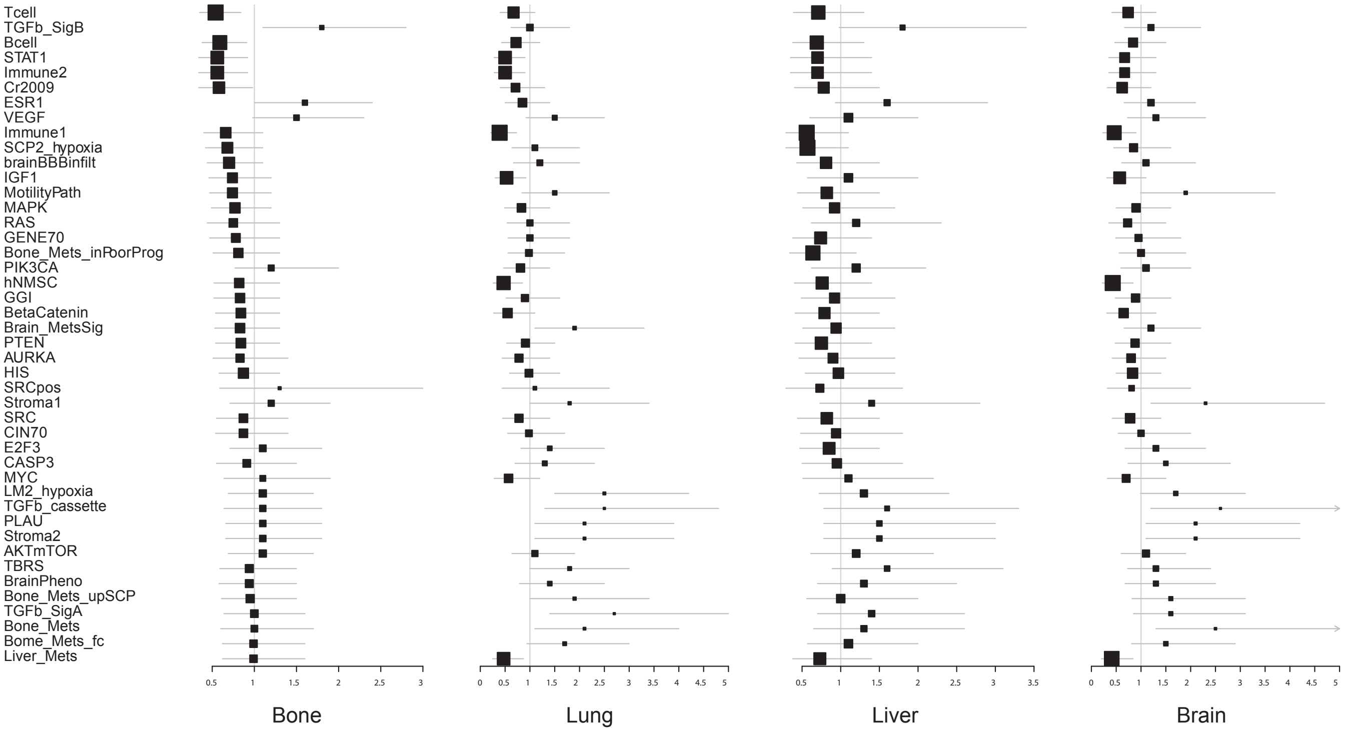

**Supplementary Figure S4. ROC curve analysis of the BV score.** ROC curves for BV score in the case-control ER positive pairs from GWDb, shown for each of the metastatic groups versus all other events, respectively. The plot for 'bone & visceral' versus 'no metastasis' uses the BV discovery set. In the 'visceral only' and 'bone only' plots, the events in each metastatic group were not used during BV discovery.

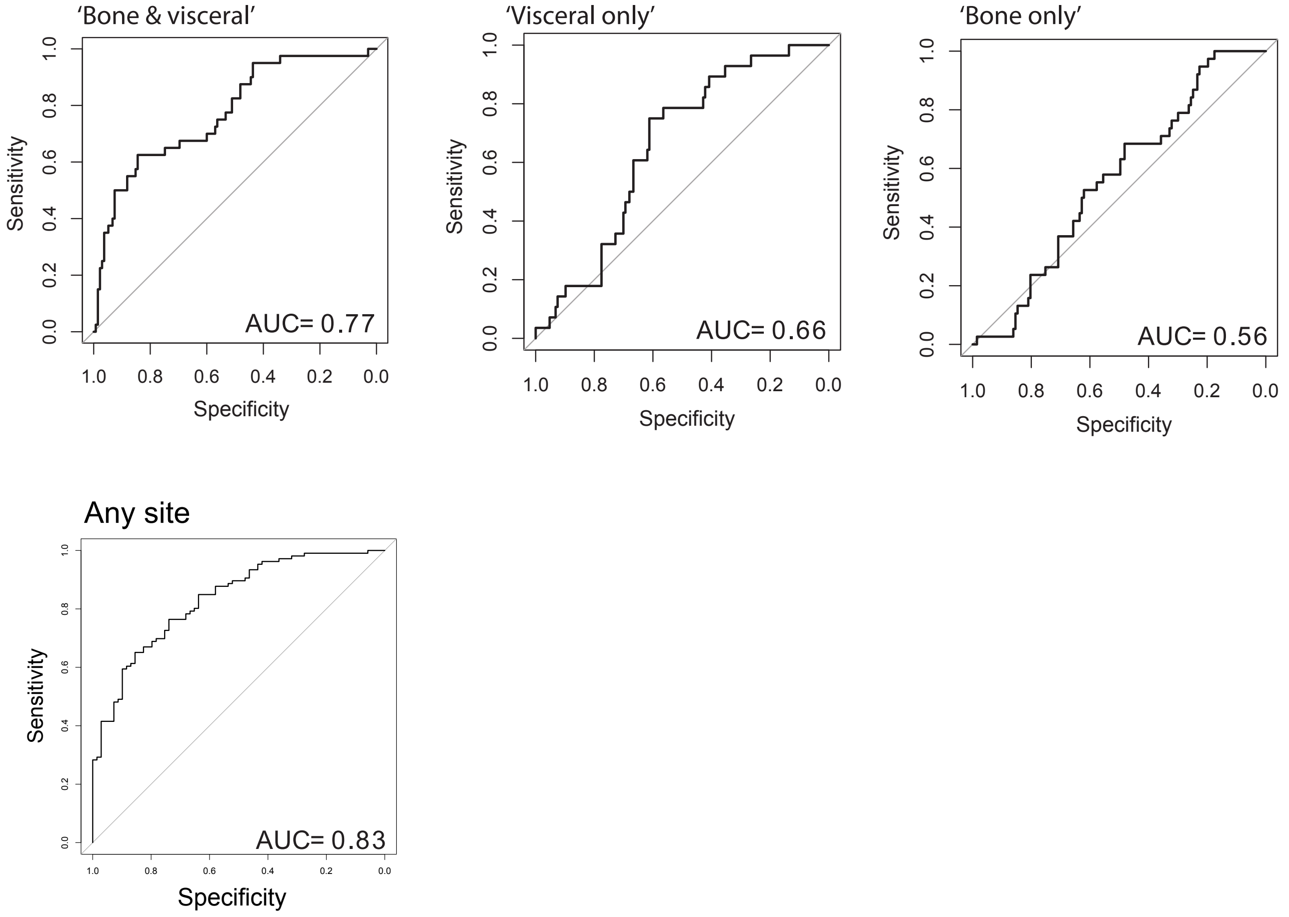

**Supplementary Figure S5. RNA diagnostics from FFPE samples: distributions and relation to sample age, fixative type. A-D.** Number of primary tumour (**A**) and lymph node metastasis (**B**) RNA samples shown by year of diagnosis and fixative type. RNA yield (Nanodrop) from primary tumour samples (**C**) and lymph node metastasis samples (**D**) shown by year of diagnosis. The median yield is indicated (horizontal line). **E.** A260/A280 ratio for primary tumour samples. The ratio A260/A280=2 is indicated (horizontal line). **F.** A260/A280 ratio for lymph node metastasis samples. **G.** Ct values for primary tumour RNA samples (dotted line, Ct = 29). **H.** Ct values for lymph node metastasis samples. **I.** Comparison of RNA yield with tumour characteristics for each decade of diagnosis (left-right); top row: samples are grouped according whether the sample is from lymph node metastasis sample or from a primary tumour of Grade 1, 2, 3; middle row: ER status for samples grouped according to lymph node metastasis (with the IHC ER status of the matching primary tumour ('primary, ERpos': ER-positive; 'primary, ERneg': ER-negative)) and primary tumour samples; bottom row: hormone receptor status ('HRpos': at least one of IHC ER, PgR, HER2 status is positive; 'TNBC': IHC ER-, PgR- and HER2-negative). There are no lymph node metastases with patient-matched primary tumours with known TNBC status (IHC ER-, PgR- and HER2-negative). **J.** Comparison of Ct values with tumour characteristics for each decade of diagnosis (left-right) and primary tumour characteristics (top-bottom, as for (**I**)). All boxplots show median, interquartile range (IQR) and outliers (points, >1.5 IQR). Notches indicate ±1.58 IQR/√n, where shown.

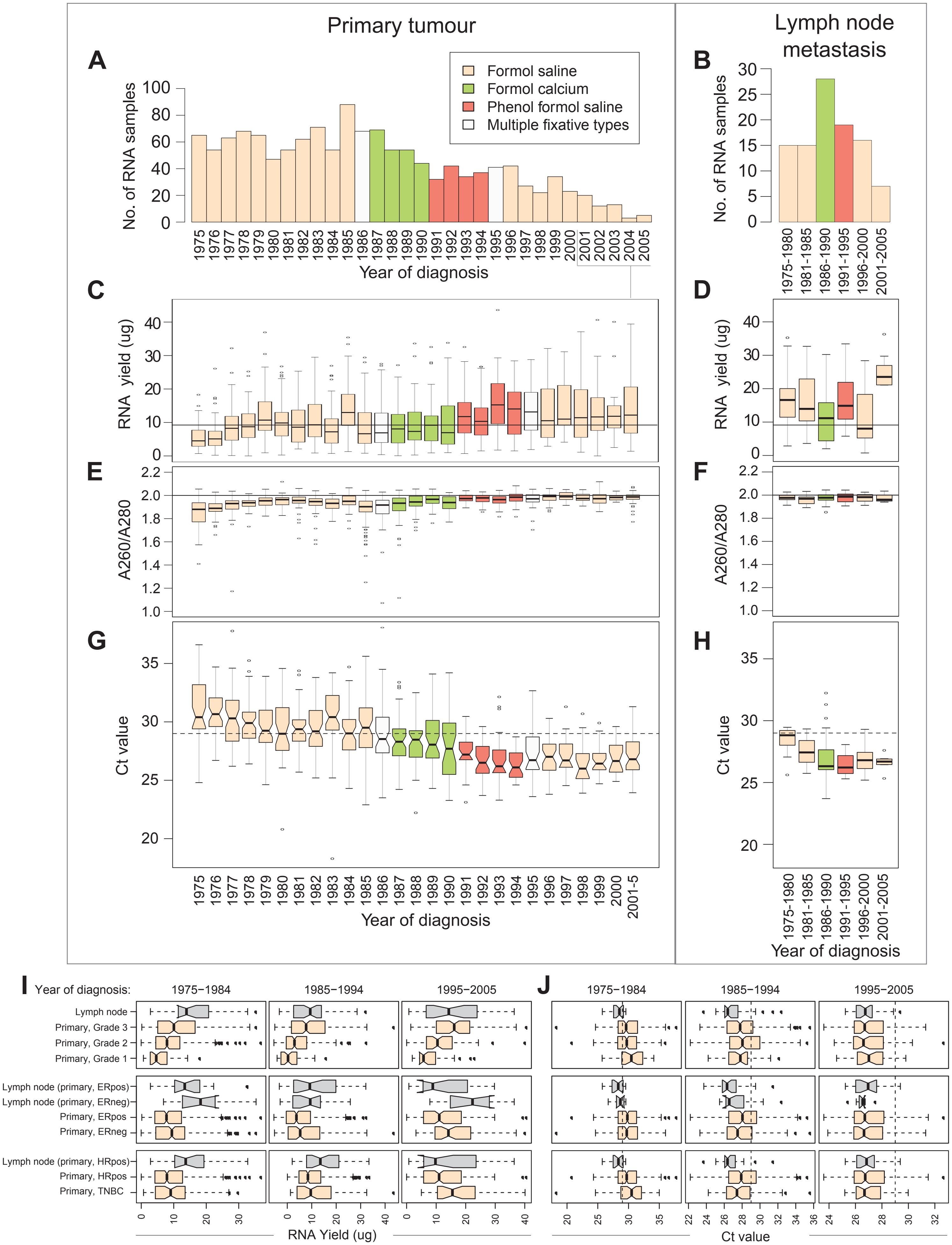
Primary tumour
Lymph node metastasis
A
B
C
D
E
F
G
H
I
J
Formol saline
Formol calcium
Phenol formol saline
Multiple fixative types
No. of RNA samples
Year of diagnosis
RNA yield (ug)
A260/A280
Ct value
Year of diagnosis:
1975–1984
1985–1994
1995–2005
Lymph node
Primary, Grade 3
Primary, Grade 2
Primary, Grade 1
Lymph node (primary, ERpos)
Lymph node (primary, ERneg)
Primary, ERpos
Primary, ERneg
Lymph node (primary, HRpos)
Primary, HRpos
Primary, TNBC
RNA Yield (ug)
Ct value

**Supplementary Figure S6.** Primary tumour and lymph node metastasis samples assayed on plates 8-15: RNA diagnostics compared with WG-DASL data quality. **A.** Primary tumour samples assayed using WG-DASL on plates 8-15 (n=574) shown by year of surgery, and therefore related to the age of the FFPE tissue block. Samples with Ct < 29 were prioritised for WG-DASL and samples with Ct > 29 were also included (light blue: Ct < 29; dark blue: Ct > 29). Selection of samples for WG-DASL was based on Ct value only, within each extraction batch and without reference to any other sample factors. On inspection, the full range of tissue block ages (year of surgery, 1975-2005) is represented amongst the assayed RNA samples. **B.** Lymph node metastasis samples assayed using WG-DASL (n=79). On inspection, the full range of tissue block ages is represented. **C,D.** Ct values of assayed primary tumour (C) and lymph node metastasis samples (D) vary by year of surgery, reflecting the variation by storage time for all samples. **E.** Higher Pearson correlation of technical duplicates is associated with overall lower Ct value and higher RNA yield, but is not associated with the density of fragments with length 150nt to 250nt. Pearson correlation of technical duplicates was calculated using quantile normalised data. **F (inset).** Density plot of pairwise Pearson correlation coefficients, shown for pairs of technical duplicates (line plot and rug). **G.** Array median of log2 raw intensity values with lowess smoother (bold green) showing a modest association between higher Ct values and lower array intensities. Smoothed array mean (bold red) and global median (dotted green) are also shown. Ct values for eleven failed arrays (no data) are indicated (red crosses). **H.** Distribution of (i) Ct values, (ii)-(iv) density of RNA fragments within the specified nucleotide length, shown according to array data quality. 'POOR': array failure/lower quality (defined here as outlying samples satisfying median log2(intensity) < 8.5, or standard deviation of log2(intensity) < 1.8, or fewer than 18,000 probes with detection p-value < 0.01). 'OK': all other arrays.

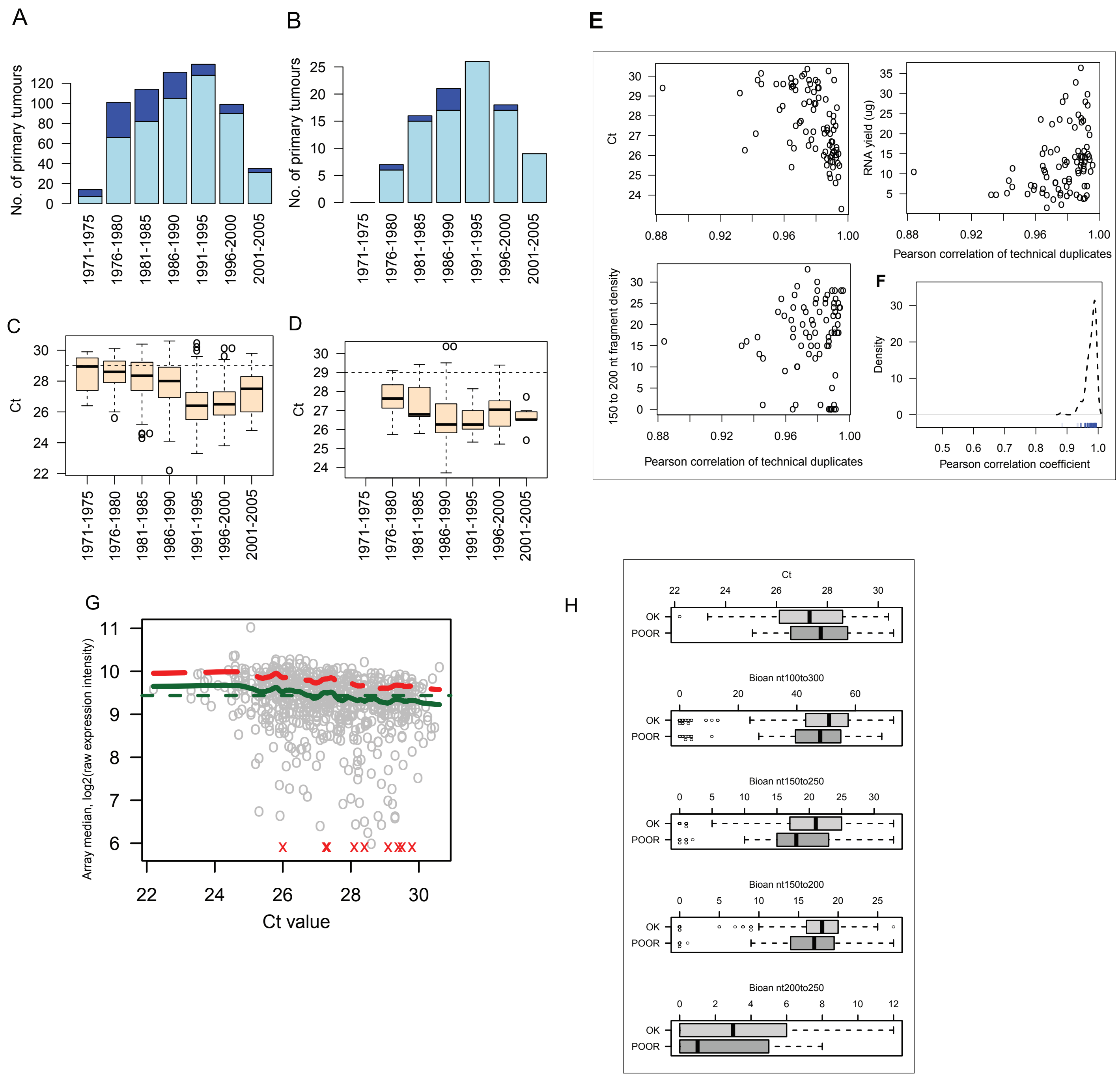

**Supplementary Figure S7.** Breast cancer cell line expression profiles. Unsupervised hierarchical clustering of the 300 most variable probes (by standard deviation) across cell line samples. All duplicated cell line arrays cluster together regardless of hybridisation plate/BeadChip. The two arms of the sample (column) dendrogram correspond to basal-like (blue branches) and luminal-like cell lines (pink), respectively (Basal-like: MD231, HCC1954, HCC1143; Luminal-like: T47D, MCF7, BT474 (Neve, Chin et al. 2006)). The probe dendrogram (rows) displayed breast cancer cell linespecific expression profiles as previously reported (Neve, Chin et al. 2006; Grigoriadis, Mackay et al. 2012).

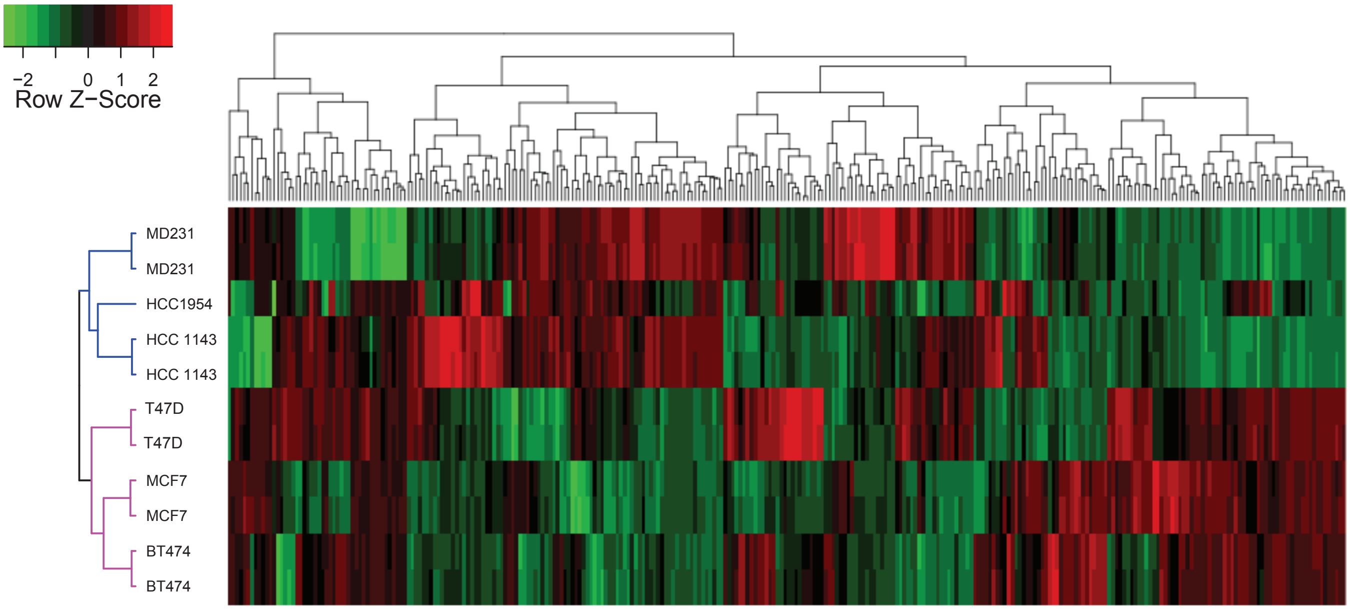

**Supplementary Figure S8.** Annotation enrichment (StringDB [http://string-db.org/]) of the candidate BV gene module. **A.** GO annotation network of *BV* genes. Red nodes indicate genes annotated as “GO: 0000793 condensed chromosome”. **B.** Table of top-ranked enriched GO annotation.

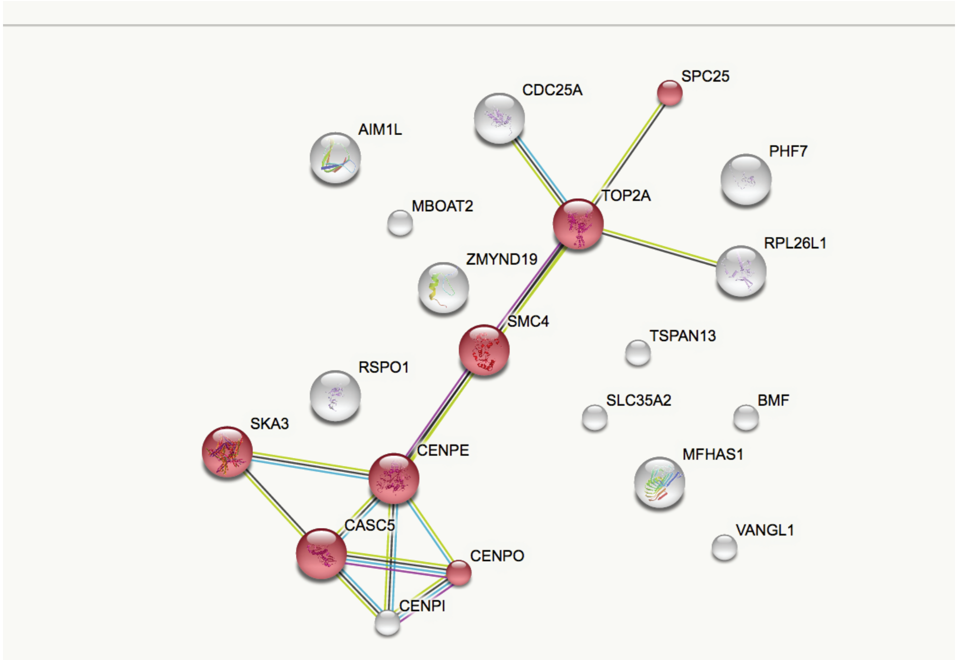


**Biological Process (GO)**

| pathway ID | pathway description | count in gene set | false discovery rate |
|---|---|---|---|
| GO:0000278 | mitotic cell cycle | 9 | 0.000228 |
| GO:0000280 | nuclear division | 7 | 0.000382 |
| GO:0034508 | centromere complex assembly | 4 | 0.000382 |
| GO:0051301 | cell division | 7 | 0.000391 |
| GO:0007067 | mitotic nuclear division | 6 | 0.00118 |
| | | | *(more ...)* |

**Cellular Component (GO)**

| pathway ID | pathway description | count in gene set | false discovery rate |
|---|---|---|---|
| GO:0000793 | condensed chromosome | 7 | 3.17e-07 |
| GO:0000776 | kinetochore | 6 | 7.8e-07 |
| GO:0000775 | chromosome, centromeric region | 6 | 4.18e-06 |
| GO:0000777 | condensed chromosome kinetochore | 5 | 9.65e-06 |
| GO:0000779 | condensed chromosome, centromeric region | 5 | 9.65e-06 |
