# Supplementary material for: Gene expression modules in primary breast cancers as risk factors for organotropic patterns of first metastatic spread: a case control study
Source: Breast Cancer Res. 2017 Oct 13;19:113. doi: 10.1186/s13058-017-0881-y (PMC5640935; doi:10.1186/s13058-017-0881-y)
Supplement: Supplementary file 4 — Estimated ORs for molecular subtype representation based on conditional logistic regression. (PDF 1 mb) [file 13058_2017_881_MOESM4_ESM.pdf]

Table 3

V SERIES

		Cases	Controls	n, paired: Cases	n, paired: Controls	n, informative: Cases	n, informative: Controls	OR (95% CI)	p
Extracted RNA									
**ER IHC**	**ER-**	99	76	*99*	*76*	*69*	*46*	Ref.	
	**ER+**	147	170	*147*	*170*	*46*	*69*	0.67 (0.46-0.97)	0.03
	**Total**	246	246	*246*	*246*	*115*	*115*		
**IHC subtypes**	**ER+HER2-**	68	70	*42*	*49*	*22*	*29*	Ref.	
	**ER+HER2+**	23	25	*11*	*17*	*11*	*17*	0.8 (0.36-1.8)	0.6
	**ER-HER2+**	35	12	*16*	*8*	*15*	*7*	2.2 (0.88-5.7)	0.09
	**TNBC**	36	31	*24*	*19*	*20*	*15*	1.4 (0.69-2.9)	0.3
Not assigned (missing IHC data)		*84*	*108*						
	**Total**	246	246	*93*	*93*	*68*	*68*		
GWDb									
**PAM50**	**Luminal A**	21	34	*10*	*20*	*7*	*17*	Ref.	
	**Luminal B**	17	9	*10*	*9*	*6*	*5*	2.7 (0.62-11)	0.2
	**Basal**	28	17	*11*	*10*	*11*	*10*	1.7 (0.62-4.7)	0.3
	**HER2**	30	15	*15*	*6*	*11*	*2*	8.6 (1.6-46)	0.01
	**Normal**	9	11	*5*	*6*	*4*	*5*	1.6 (0.35-7.4)	0.5
Not assigned (missing from GWDb)		*141*	*160*						
	**Total**	*246*	*246*	*51*	*51*	*39*	*39*		
**IntClust, grouped**	**3**	9	21	*6*	*11*	*5*	*10*	Ref.	
	**1,6,9**	20	8	*11*	*6*	*10*	*5*	3.8 (0.75-20)	0.1
	**2,4**	24	18	*13*	*10*	*10*	*7*	2.6 (0.70-9.5)	0.2
	**5**	20	6	*9*	*1*	*9*	*1*	17 (1.6-188)	0.02
	**7,8**	12	22	*4*	*16*	*4*	*16*	0.66 (0.15-2.9)	0.6
	**10**	20	11	*8*	*7*	*8*	*7*	2.1 (0.47-9.3)	0.3
Not assigned (missing from GWDb)		*141*	*160*						
	**Total**	*246*	*246*	*51*	*51*	*46*	*46*		
**IntClust**	**3**	9	21	*6*	*11*	*5*	*10*		
	**1**	12	0	*8*	*0*	*8*	*0*		
	**2**	6	4	*4*	*4*	*4*	*4*		
	**4**	18	14	*9*	*6*	*9*	*6*		
	**5**	20	6	*9*	*1*	*9*	*1*		
	**6**	4	1	*2*	*1*	*2*	*1*		
	**7**	5	13	*1*	*10*	*1*	*10*		
	**8**	7	9	*3*	*6*	*3*	*6*		
	**9**	4	7	*1*	*5*	*1*	*5*		
	**10**	20	11	*8*	*7*	*8*	*7*		
Not assigned (missing from GWDb)		*141*	*160*						
	**Total**	*246*	*246*	*51*	*51*	*50*	*50*		

BV SERIES

		Cases	Controls	n, paired: Cases	n, paired: Controls	n, informative: Cases	n, informative: Controls	OR (95% CI)	p
Extracted RNA									
**ER IHC**	**ER-**	96	74	*96*	*74*	*67*	*45*	Ref.	
	**ER+**	162	184	*162*	*184*	*45*	*67*	0.67 (0.46-0.98)	0.04
	**Total**	258	258	*258*	*258*	*112*	*112*		
**IHC subtypes**	**ER+HER2-**	78	74	*48*	*55*	*15*	*22*	Ref.	
	**ER+HER2+**	25	17	*12*	*12*	*10*	*10*	1.3 (0.48-3.3)	0.7
	**ER-HER2+**	30	13	*17*	*9*	*16*	*8*	2.2 (0.87-5.3)	0.1
	**TNBC**	33	25	*18*	*19*	*12*	*13*	1.1 (0.48-2.7)	0.8
Not assigned (missing IHC data)		*92*	*129*						
	**Total**	258	258	*95*	*95*	*53*	*53*		
GWDb									
**PAM50**	**Luminal A**	33	45	*19*	*25*	*10*	*16*	Ref.	
	**Luminal B**	26	13	*13*	*5*	*12*	*4*	2.5 (0.79-8.1)	0.1
	**Basal**	21	13	*14*	*9*	*7*	*2*	3.0 (0.61-15)	0.2
	**HER2**	20	12	*4*	*9*	*2*	*7*	0.4 (0.081-2.1)	0.3
	**Normal**	6	7	*1*	*3*	*1*	*3*	0.5 (0.050-5.4)	0.6
Not assigned (missing from GWDb)		*152*	*168*						
	**Total**	258	258	*51*	*51*	*32*	*32*		
**IntClust, grouped**	**3**	14	25	*9*	*14*	*8*	*13*	Ref.	
	**1,6,9**	26	13	*11*	*7*	*10*	*6*	2.5 (0.71-9.1)	0.2
	**2,4**	14	18	*6*	*9*	*3*	*6*	0.86 (0.18-4.1)	0.9
	**5**	17	3	*4*	*1*	*4*	*1*	7.3 (0.66-81)	0.1
	**7,8**	22	23	*12*	*13*	*10*	*11*	1.4 (0.51-3.9)	0.5
	**10**	13	8	*9*	*7*	*5*	*3*	2.2 (0.43-10.7)	0.4
Not assigned (missing from GWDb)		*152*	*168*						
	**Total**	258	258	*51*	*51*	*40*	*40*		
**IntClust**	**3**	14	25	*9*	*14*	*8*	*13*		
	**1**	9	6	*4*	*4*	*4*	*4*		
	**2**	4	1	*2*	*0*	*2*	*0*		
	**4**	10	17	*4*	*9*	*3*	*8*		
	**5**	17	3	*4*	*1*	*4*	*1*		
	**6**	7	2	*4*	*1*	*4*	*1*		
	**7**	9	11	*5*	*5*	*4*	*4*		
	**8**	13	12	*7*	*8*	*7*	*8*		
	**9**	10	5	*3*	*2*	*2*	*1*		
	**10**	13	8	*9*	*7*	*5*	*3*		
Not assigned (missing from GWDb)		*152*	*168*						
	**Total**	258	258	*51*	*51*	*43*	*43*		

B SERIES

		Cases	Controls	n, paired: Cases	n, paired: Controls	n, informative: Cases	n, informative: Controls	OR (95% CI)	p
Extracted RNA									
**ER IHC**	**ER-**	50	68	*50*	*68*	*32*	*50*	Ref.	
	**ER+**	188	170	*188*	*170*	*50*	*32*	1.6 (1.0-2.4)	0.05
	**Total**	238	238	*238*	*238*	*50*	*50*		
**IHC subtypes**	**ER+HER2-**	86	84	*56*	*48*	*21*	*13*	Ref.	
	**ER+HER2+**	22	15	*13*	*11*	*10*	*8*	1.1 (0.43-3.0)	0.8
	**ER-HER2+**	13	11	*7*	*8*	*4*	*5*	0.84 (0.22-3.2)	0.8
	**TNBC**	14	23	*5*	*14*	*3*	*12*	0.25 (0.07-0.89)	0.03
Not assigned (missing IHC data)		*103*	*105*						
	**Total**	238	238	*81*	*81*	*38*	*38*		
GWDb									
**PAM50**	**Luminal A**	32	45	*16*	*25*	*9*	*18*	Ref.	
	**Luminal B**	28	16	*15*	*6*	*14*	*5*	4.2 (1.3-14)	0.02
	**Basal**	9	17	*6*	*10*	*4*	*8*	0.66 (0.19-2.3)	0.5
	**HER2**	18	12	*6*	*4*	*5*	*3*	2.9 (0.57-15)	0.2
	**Normal**	11	8	*7*	*5*	*6*	*4*	2.5 (0.55-11)	0.2
Not assigned (missing from GWDb)		*140*	*140*						
	**Total**	238	238	*50*	*50*	*38*	*38*		
**IntClust, grouped**	**3**	19	25	*11*	*13*	*8*	*10*	Ref.	
	**1,6,9**	18	17	*11*	*8*	*11*	*8*	2.0 (0.60-6.6)	0.3
	**2,4**	26	14	*12*	*7*	*10*	*5*	2.1 (0.49-8.6)	0.3
	**5**	7	5	*3*	*1*	*3*	*1*	5.1 (0.43-61)	0.2
	**7,8**	24	27	*10*	*14*	*6*	*10*	0.89 (0.25-3.1)	0.9
	**10**	4	10	*3*	*7*	*3*	*7*	0.59 (0.13-2.7)	0.5
Not assigned (missing from GWDb)		*140*	*140*						
	**Total**	238	238	*50*	*50*	*41*	*41*		
**IntClust**	**3**	19	25	*11*	*13*	*8*	*10*		
	**1**	5	13	*3*	*7*	*3*	*7*		
	**2**	8	3	*4*	*2*	*4*	*2*		
	**4**	18	11	*8*	*5*	*6*	*3*		
	**5**	7	5	*3*	*1*	*3*	*1*		
	**6**	5	2	*3*	*0*	*3*	*0*		
	**7**	17	14	*4*	*8*	*3*	*7*		
	**8**	7	13	*6*	*6*	*5*	*5*		
	**9**	8	2	*5*	*1*	*5*	*1*		
	**10**	4	10	*3*	*7*	*3*	*7*		
Not assigned (missing from GWDb)		*140*	*140*						
	**Total**	238	238	*50*	*50*	*43*	*43*		

ANY CASE TYPE

		Cases	Controls	n, paired: Cases	n, paired: Controls	n, informative: Cases	n, informative: Controls	OR (95% CI)	p
Extracted RNA									
**ER IHC**	**ER-**	245	218	*245*	*218*	*168*	*141*	Ref.	
	**ER+**	497	524	*497*	*524*	*141*	*168*	0.84 (0.67-1.0)	0.1
	**Total**	742	742	*742*	*742*	*309*	*309*		
**IHC subtypes**	**ER+HER2-**	232	228	*146*	*152*	*58*	*64*	Ref.	
	**ER+HER2+**	70	57	*36*	*40*	*31*	*35*	0.96 (0.58-1.6)	0.9
	**ER-HER2+**	78	36	*40*	*25*	*35*	*20*	1.7 (0.97-3.0)	0.07
	**TNBC**	83	79	*47*	*52*	*35*	*40*	0.93 (0.58-1.5)	0.8
Not assigned (missing IHC data)		*279*	*342*						
	**Total**	742	742	*269*	*269*	*159*	*159*		
GWDb									
**PAM50**	**Luminal A**	86	124	*45*	*70*	*26*	*51*	Ref.	
	**Luminal B**	71	38	*38*	*20*	*32*	*14*	2.9 (1.5-5.9)	0.002
	**Basal**	58	47	*31*	*29*	*22*	*20*	1.4 (0.7-2.7)	0.3
	**HER2**	68	39	*25*	*19*	*18*	*12*	2.3 (1.0-5.0)	0.05
	**Normal**	26	26	*13*	*14*	*11*	*12*	1.4 (0.6-3.5)	0.4
Not assigned (missing from GWDb)		433	468						
	**Total**	742	742	*152*	*152*	*109*	*109*		
**IntClust, grouped**	**3**	42	71	*26*	*38*	*21*	*33*	Ref.	
	**1,6,9**	64	38	*33*	*21*	*31*	*19*	2.6 (1.2-5.6)	0.01
	**2,4**	64	50	*31*	*26*	*23*	*18*	1.9 (0.88-4.2)	0.1
	**5**	44	14	*16*	*3*	*16*	*3*	9.0 (2.3-35)	0.002
	**7,8**	58	72	*26*	*43*	*20*	*37*	0.95 (0.49-1.9)	0.9
	**10**	37	29	*20*	*21*	*16*	*17*	1.5 (0.64-3.3)	0.4
Not assigned (missing from GWDb)		*433*	*468*						
	**Total**	742	742	*152*	*152*	*127*	*127*		
**IntClust**	**3**	42	71	*26*	*38*	*21*	*33*	Ref.	
	**1**	26	19	*15*	*11*	*15*	*11*	2.3 (0.88-6.2)	0.09
	**2**	18	8	*10*	*6*	*10*	*6*	3.0 (0.90-10)	0.07
	**4**	46	42	*21*	*20*	*18*	*17*	1.5 (0.67-3.5)	0.3
	**5**	44	14	*16*	*3*	*16*	*3*	8.7 (2.2-34)	0.002
	**6**	16	5	*9*	*2*	*9*	*2*	6.0 (1.2-31)	0.03
	**7**	31	38	*10*	*23*	*8*	*21*	0.63 (0.25-1.6)	0.3
	**8**	27	34	*16*	*20*	*15*	*19*	1.3 (0.56-2.8)	0.6
	**9**	22	14	*9*	*8*	*8*	*7*	1.8 (0.56-5.7)	0.3
	**10**	37	29	*20*	*21*	*16*	*17*	1.4 (0.61-3.1)	0.4
Not assigned (missing from GWDb)		*433*	*468*						
	**Total**	742	742	*152*	*152*	*136*	*136*		

Footnotes: Conditional logistic regression.
Epi::clogistic, Case_Control ~ x + strata(Random_Selection)
Note: Counts of pairs (not missing) and informative entries in the conditional logistic model are shown for information.
