# Supplementary material for: Gene expression modules in primary breast cancers as risk factors for organotropic patterns of first metastatic spread: a case control study
Source: Breast Cancer Res. 2017 Oct 13;19:113. doi: 10.1186/s13058-017-0881-y (PMC5640935; doi:10.1186/s13058-017-0881-y)
Supplement: Supplementary file 5 — Estimated ORs for molecular subtype representations based on logistic regression. (PDF 657 kb) [file 13058_2017_881_MOESM5_ESM.pdf]

Table 4

		V SERIES		n, uniq			BV SERIES		n, uniq			B SERIES		n, uniq			ANY CASE TYPE		n, uniq		
		Cases	Controls	Controls	OR (95% CI)	p	Cases	Controls	Controls	OR (95% CI)	p	Cases	Controls	Controls	OR (95% CI)	p	Cases	Controls	Controls	OR (95% CI)	p
Extracted RNA																					
**ER IHC**	**ER-**	99	76	72	Ref.		96	74	71	Ref.		50	68	64	Ref.		245	218	184	Ref.	
	**ER+**	147	170	160	0.67 (0.46-0.97)	0.04	162	184	174	0.69 (0.47-1.0)	0.05	188	170	158	1.5 (1.0-2.3)	0.05	497	524	436	0.86 (0.68-1.1)	0.2
	**Total**	**246**	**246**	**232**			**258**	**258**	**245**			**238**	**238**	**222**			**742**	**742**	**620**		
**IHC subtypes**	**ER+HER2-**	68	70	67	Ref.		78	74	72	Ref.		86	84	80	Ref.		232	228	196	Ref.	
	**ER+HER2+**	23	25	23	0.99 (0.50-1.9)	1.0	25	17	17	1.4 (0.68-2.8)	0.4	22	15	14	1.5 (0.71-3.1)	0.3	70	57	43	1.4 (0.90-2.1)	0.1
	**ER-HER2+**	35	12	12	2.9 (1.4-6.2)	0.005	30	13	13	2.1 (1.0-4.5)	0.04	13	11	9	1.3 (0.55-3.4)	0.5	78	36	31	2.1 (1.4-3.4)	0.001
	**TNBC**	36	31	30	1.2 (0.66-2.1)	0.6	33	25	23	1.3 (0.71-2.5)	0.4	14	23	23	0.57 (0.27-1.2)	0.1	83	79	68	1.0 (0.71-1.5)	0.9
	**Total**	**162**	**138**	**132**			**166**	**129**	**125**			**135**	**133**	**126**			**463**	**400**	**338**		
Not assigned (missing IHC data)		*84*	*108*	*100*			*92*	*129*	*120*			*103*	*105*	*96*			*279*	*342*	*282*		
Total (extracted RNA)		*246*	*246*	*232*			*258*	*258*	*245*			*238*	*238*	*222*			*742*	*742*	*620*		
GWDb																					
**PAM50**	**Luminal A**	21	34	33	Ref.		33	45	42	Ref.		32	45	42	Ref.		86	124	109	Ref.	
	**Luminal B**	17	9	9	3.0 (1.1-8.2)	0.03	26	13	12	2.7 (1.2-6.4)	0.02	28	16	15	2.5 (1.1-5.4)	0.02	71	38	35	2.6 (1.6-4.2)	0.0002
	**Basal**	28	17	15	2.9 (1.3-6.9)	0.01	21	13	13	2.1 (0.9-4.8)	0.09	9	17	16	0.74 (0.28-1.9)	0.5	58	47	40	1.8 (1.1-3.0)	0.02
	**HER2**	30	15	14	3.4 (1.5-8.0)	0.005	20	12	12	2.1 (0.92-5.1)	0.08	18	12	10	2.4 (0.98-6.0)	0.06	68	39	33	2.6 (1.6-4.4)	<1e-4
	**Normal**	9	11	11	1.3 (0.45-3.6)	0.6	6	7	7	1.1 (0.32-3.6)	0.9	11	8	7	2.1 (0.7-6.2)	0.2	26	26	23	1.4 (0.76-2.7)	0.3
	**Total**	**105**	**86**	**82**			**106**	**90**	**86**			**98**	**98**	**90**			**309**	**274**	**240**		
Not assigned (not in GWDb)		*141*	*160*	*150*			*152*	*168*	*159*			*140*	*140*	*132*			*433*	*468*	*380*		
Total (extracted RNA)		*246*	*246*	*232*			*258*	*258*	*245*			*238*	*238*	*222*			*742*	*742*	*620*		
**IntClust, grouped**	**3**	9	21	20	Ref.		14	25	25	Ref.		19	25	23	Ref.		42	71	66	Ref.	
	**1,6,9**	20	8	8	5.6 (1.8-18)	0.003	26	13	12	3.9 (1.5-10)	0.01	18	17	15	1.5 (0.45-1.5)	0.4	64	38	35	2.9 (1.6-5.1)	0.0003
	**2,4**	24	18	17	3.1 (1.2-8.9)	0.03	14	18	17	1.5 (0.6-3.9)	0.4	26	14	13	2.4 (1.0-6.1)	0.05	64	50	42	2.4 (1.4-4.2)	0.002
	**5**	20	6	5	8.9 (2.7-34)	0.001	17	3	3	10 (2.8-49)	0.001	7	5	4	2.1 (0.55-9.1)	0.3	44	14	11	6.3 (3.0-14)	<1e-4
	**7,8**	12	22	21	1.3 (0.44-3.7)	0.7	22	23	21	1.9 (0.78-4.6)	0.2	24	27	26	1.1 (0.49-2.6)	0.8	58	72	59	1.5 (0.91-2.6)	0.1
	**10**	20	11	11	4.0 (1.4-12)	0.01	13	8	8	2.9 (0.99-9.0)	0.06	4	10	9	0.54 (0.13-1.9)	0.4	37	29	27	2.2 (1.2-4.1)	0.02
	**Total**	**105**	**86**	**82**			**106**	**90**	**86**			**98**	**98**	**90**			**309**	**274**	**240**		
Not assigned (not in GWDb)		*141*	*160*	*150*			*152*	*168*	*159*			*140*	*140*	*132*			*433*	*468*	*380*		
Total (extracted RNA)		*246*	*246*	*232*			*258*	*258*	*245*			*238*	*238*	*222*			*742*	*742*	*620*		

Footnotes: Logistic regression model.

stats::glm, Case_Control ~ x, family='binomial'

Note: This is an unpaired test. A small number of patients were duplicated within a control series by design; here, patients were de-duplicated within each control series ("n, uniq"). There are no duplicates within each case series by design. Duplicates were permitted between cases and controls. Entries with missing values in the independent variable were excluded.
